# FEZF1-AS1 drives autophagy-mediated progression of colon cancer and reduces chemosensitivity through inhabiting the PI3K/AKT/mTOR signaling pathway

**DOI:** 10.3389/fgene.2025.1514205

**Published:** 2025-07-24

**Authors:** Xiaoping Yang, Zuohui Yuan, Lingzhu Gou, Long Cheng, Zirui Wang, Pingfan Wu, Xiaochun Wang, Xueni Ma, Tiantian Ma, Yi Yu, Zhiping Wu, Dekui Zhang

**Affiliations:** ^1^ Key Laboratory of Digestive Diseases of Gansu Province, The Second Hospital and Clinical Medical School, Lanzhou University, Lanzhou, China; ^2^ Department of Gastroenterology, Gansu provincial hospital, Lanzhou, China; ^3^ Department of Urology, The Second People’s Hospital of Lanzhou, Lanzhou, China; ^4^ Department of Pathology, The Sixth Affiliated Hospital, School of Medicine, South China University of Technology, Foshan, China; ^5^ Department of Pneumology, Xi An Chest Hospital, Xian, China; ^6^ Department of Gastroenterology, The Second Hospital and Clinical Medical School, Lanzhou University, Lanzhou, China

**Keywords:** FEZF1-AS1, colon cancer, autophagy, PI3K/AKT/mTOR, chemosensitivity

## Abstract

The pathogenesis and chemoresistance mechanisms of colon cancer (CC) are still unclear. Here, we find that a long non-coding RNA (lncRNA), FEZ family zinc finger 1-antisense RNA 1 (FEZF1-AS1), is highly expressed in CC, which may be caused by the amplification mutation of FEZF1-AS1 at the gene level through bioinformatic analysis. FEZF1-AS1 has the potential to be a biomarker in the diagnosis of CC. Functionally, FEZF1-AS1 promotes the proliferation, invasion, metastasis, and survival of CC cells and reduces the sensitivity of CC cells to oxaliplatin. Mechanistically, FEZF1-AS1 drives autophagy-mediated development of CC and reduces chemosensitivity to oxaliplatin through inhabiting the PI3K/AKT/mTOR signaling pathway. In summary, our data suggest that FEZF1-AS1 may be a key driver of CC progression and chemotherapy resistance, and targeting FEZF1-AS1 may be a potential strategy for the diagnosis and treatment of CC.

## Introduction

Colon cancer (CC) is one of the most common malignancies in the world and a leading cause of tumor-related death (Brenner et al., 2014; Siegel et al., 2023). In 2020, an estimated 1.148 million new cases of CC and approximately 577,000 deaths were reported globally, with a standardized incidence of 11.4 per 100,000 and a standardized mortality of 5.4 per 100,000 (Mengyuan, 2023). In terms of pathogenesis, CC is mainly divided into sporadic CC and inflammation-related CC, and there are multiple gene deletions or mutations (Nagao-Kitamoto et al., 2022)^.^ At present, the therapies for CC mainly include the following: endoscopic therapy, conventional surgery, neoadjuvant therapy, chemotherapy, targeted therapy, and immunotherapy (Wu et al., 2020; Wang et al., 2020; Georgieva et al., 2021; Du et al., 2021; Lopes et al., 2021; Wu et al., 2021; Ding et al., 2021). Despite significant advancements in the diagnosis and treatment of CC, over 10% of patients are diagnosed at an advanced stage, and nearly 30% of those initially diagnosed at an early stage eventually develop distant metastasis (Böckelman et al., 2015) and chemotherapy resistance, leading to a poor prognosis for CC patients (Wolpin et al., 2007; Mayer, 2009; Corley et al., 2017; Wolf et al., 2018). Current research works on targeted therapies and biomarker-driven therapies for CC are not promising. Therefore, it is urgent to search for key biomarkers of CC diagnosis, elucidate the molecular mechanism of CC development, and develop more effective treatments.

Long non-coding RNA (lncRNA) is an endogenous RNA with a length of >200 bases that performs regulatory functions in the human body, such as maintaining genome stability, regulating epigenetics, regulating cell differentiation, and participating in the development of human tumors (Louro et al., 2009; Gibb et al., 2011; Mercer et al., 2009; Ezkurdia et al., 2014; Zhang S. et al., 2017). FEZ family zinc finger 1-antisense RNA 1 (FEZF1-AS1) is an antisense lncRNA with a length of 2,653 bp. Because the first exon of FEZF1-AS1 contains 611 nucleotides complementary to the first exon of FEZF1 messenger RNA (mRNA), it is named FEZF1-AS1 (Shi et al., 2019). Ding et al. (2025) confirmed that FEZF1-AS1 regulated the growth of CC cells through the P53 signaling pathway and inhibited epithelial–mesenchymal transition, thereby providing new insights into potential therapeutic strategies for CC. Bian et al. (2018) pointed out that the expression of FEZF1-AS1 was significantly elevated in CC, showing a correlation with the survival of CC patients. They revealed that FEZF1-AS1 regulated the STAT3 signaling pathway in CC via PKM2, promoting the occurrence and progression of CC. In addition to the above study on the direct binding of FEZF1-AS1 to the PKM2 protein, most studies still focus on FEZF1-AS1 as a competitive endogenous RNA (ceRNA), which binds to microRNAs (miRNAs) and inhibits its expression, thereby weakening the inhibitory effect of miRNAs on downstream mRNAs. This results in increased expression of downstream mRNAs, further activating downstream tumor-related signaling pathways and playing a key role in tumor development (Li et al., 2020; Yang et al., 2023). The findings collectively indicate a significant function of FEZF1-AS1 in facilitating the development of CC.

Autophagy occurs through lysosomal degradation. This process involves the recycling of undesired or impaired cellular organelles and proteins, which is essential for the regeneration of diverse precursors, sustains cellular biosynthesis and survival, and in turn maintains intracellular homeostasis (Klionsky et al., 2016; Mizushima and Levine, 2010; Cheng et al., 2017). The classic autophagy process involves a quintet of phases: initiation, nucleation (formation of phagosomes), extension and sealing (formation of autophagosomes), fusion (formation of autophagy lysosomes), degradation, and cycling (Subramani and Malhotra, 2013; Levine and Kroemer, 2008; Yang and Klionsky, 2010). Autophagy initiation requires two protein complexes, namely, VPS34/PIK3C3 and autophagy-related gene (ATG) 1/ULK1 complexes. The formation of autophagosomes may consist of two trimeric ATG16L1 complexes, namely, ATG12-ATG5-ATG16L1 and ATG16L1-P62-microtubule-associated protein 1 light chain 3 (LC3) II (Lin and Baehrecke, 2015; Glick et al., 2010), suggesting that the control of proteins associated with autophagy plays a crucial role in preserving cellular balance. In normal cells, autophagy is the “brake” that prevents the occurrence of tumors. However, once the tumor is formed, tumor cells usually use autophagy as a survival mechanism to promote their growth and acquire chemoresistance (Wilde et al., 2018; Joffre et al., 2017).

Autophagy is activated when cells are subjected to survival stress from various stressors (Paillas et al., 2012). Due to the high metabolic demands of tumor cell proliferation and the inefficient ATP production caused by aerobic glycolysis, the tumor cells are subjected to higher metabolic stress (Jin et al., 2007; Jin and White, 2007; Jin and White, 2008; Janku et al., 2011). Studies have shown that tumor cells proliferating swiftly result in an absence of healthy and mature blood vessels in solid tumors, potentially triggering the autophagy process due to subsequent microenvironmental factors like hypoxia and lack of nutrients (Brown et al., 2001; Goel et al., 2011). Tumor cells often acquire genes that inhibit apoptosis, and undergo epigenetic changes that make them more dependent on autophagy (Degenhardt et al., 2006; Lum et al., 2005). Autophagy is more prominent in oxygen-deprived areas of blood vessels at the farthest reaches of the tumor, where it acts as an alternative energy source to support the survival of tumor cells. A study has reported that a variety of intracellular signaling pathways play a role in autophagy, such as PI3K/AKT/mTOR, Wnt/β-catenin, and JUK/CJun signaling pathways (Xu et al., 2020). Gui et al. (2021) reported that FEZF1-AS1 activated autophagy by acting directly with ATG5 to promote the progression of gastric cancer. Zhai et al. (2013) showed that miR-502 inhibited the formation of autophagy by inhibiting the expression of Rab1B, which is a key protein of autophagy, thus inhibiting the growth of CC. A study has also shown that the activation of autophagy can inhibit the apoptosis of CC cells and promote their proliferation (Liu J. et al., 2019). There is increasing evidence that autophagy also plays a protective role against tumor chemotherapy, possibly by participating in chemoresistance processes (Li et al., 2010; Yan et al., 2019). Wang et al. (2021) reported that autophagy induced by the MUL1/ULK1 signaling pathway promoted CC cells to produce resistance in response to chemotherapy.

At present, little is known about the association of FEZF1-AS1 with autophagy and chemoresistance in CC. Therefore, in this study, we will focus on the expression of FEZF1-AS1 in CC, its role in CC development and autophagy-related mechanisms, and whether FEZF1-AS1 mediates autophagy to affect the chemosensitivity of CC cells. The technology roadmaps of the manuscript are shown in Supplementary Figure S1.

## Materials and methods

### Bioinformatic analysis

In the Cancer Genome Atlas (TCGA) database (2020) (https://portal.gdc.cancer.gov/) (Weinstein et al., 2013), we got 371 CC patients’ RNA sequencing (RNA-seq) data and the corresponding clinical information, encompassing 38 normal colon tissues and 387 CC tissues. We obtained differently expressed RNA as log2 fold change (logFC) using the “limma” package (Ritchie et al., 2015) in R language (Tippmann, 2015) and determined that |logFC| was >2.0, and the corrected P-value (adj. P. Val) was <0.01. The results were visualized using the “ggplot2” package (Postma and Goedhart, 2019).

We logged in to NONCODE (http://www.noncode.org/), NCBI (https://pubmed.ncbi.nlm.nih.gov/), UCSC (http://genome.ucsc.edu/), GEPIA (http://gepia.cancer-pku.cn/), and lnCAR (https://lncar.renlab.org/) databases to search the expression of FEZF1-AS1 in CC and normal colon tissues. We also logged in to the cBioPortal (www.cbioportal.org/) database to analyze the mutation of FEZF1-AS1 in CC.

We used the “clusterProfiler” package (Yu et al., 2012) to analyze the Gene Ontology (GO) and Kyoto Encyclopedia of Genes and Genomes (KEGG) functions of FEZF1-AS1 and its co-expressed differentially expressed mRNA (DEmRNA) in the TCGA database. We further studied the function of FEZF1-AS1 and its co-expressed gene set using GSEA (Subramanian et al., 2005). The “pROC” package (Robin et al., 2011) was used for the receiver operating characteristic (ROC) analysis of TCGA data, and the area under the ROC curve was defined as area under curve (AUC).

In the TCGA database (2023), COX regression was used to analyze CC-related risk factors. The effect of FEZF1-AS1 on the survival of CC patients was analyzed in the GEPIA database. The mean expression of FEZF1-AS1 in CC patients was set as the cutoff value. The Mann–Whitney U test and Kruskal–Wallis test were used to examine the relationship between the clinicopathological parameters of CC patients and FEZF1-AS1.

### Tissue collection

We collected 36 CC tissue samples from the Lanzhou University Second Hospital, and all of them were confirmed to have colonic adenocarcinoma by postoperative pathologic examination. Normal colon tissues located 5 cm beyond the tumor resection margin from patients with CC were used as control. None of the patients underwent chemotherapy or radiotherapy before surgery.

### Real-time quantitative PCR (qRT-PCR)

We used TRIzol reagent (Ambion, US) to extract total RNA from cells or frozen tumor tissues. Equal amounts of mRNAs were synthesized into cDNAs using the Evo M-MLV RT Mix Kit, along with the gDNA clean reagent (AG, China) and the PCR reverse transcriptase instrument (Heal Force, China). The SYBR Green Pro Taq HS kit reagent (AG, China) and fluorescence instrument (Bio-Rad, US) were used to conduct real-time quantitative PCR (qRT-PCR). Using β-actin as the internal reference gene, the relative gene expression was calculated using the 2^−ΔΔCT^ method: ∆∆Ct = experimental group (Ct value of the target gene − Ct value of the internal reference gene) − control group (Ct value of the target gene − Ct value of the internal reference gene). Supplementary Table S1 enumerates the primer sequences used in qRT-PCR.

### Cell culture, lentiviral transfection, and reagents

The human CC cell lines RKO and Caco-2 and the human normal colon epithelial cell line NCM460 used in this experiment were acquired from the Shanghai Cell Bank of the Chinese Academy of Sciences. Cells were cultured in RPMI 1640 (BasalMedia, China) containing 10% fetal bovine serum (FBS) (SE OU, China) in a 5% CO_2_ incubator at 37°C. Every cell line underwent verification through the STR technique and was examined for potential *mycoplasma* impurities. We commissioned HANBO (China) to construct FEZF1-AS1-overexpressing lentiviral vectors and knockdown lentiviral vectors, and then Caco-2 and RKO cells were further transfected. The design of interference targets and the synthesis of primers for target fragments are shown in Supplementary Table S2. We used chloroquine (CQ; 300 μmol/L, 12 h; Selleck) and rapamycin (RA; 0.62 nmol/L, 5 h; Selleck) to inhibit or activate autophagy. Oxaliplatin (Oxa; 50 μmol/L, 20 μmol/L; Selleck) was used to further observe cell apoptosis.

### Cell Counting Kit 8 (CCK8) Assay

Cells were collected, digested, and counted, and their density was adjusted to 1 × 10^4^ cells/well. The 96-well plates were placed in a cell incubator. After culturing for 24 h, 48 h, and 72 h, the culture medium in the 96-well plate was discarded; then, 100 μL of 10% Cell Counting Kit-8 (CCK8; Beyotime, China) was added and incubated in the cell incubator for 2 h. The cell proliferation curve was drawn based on absorbance (OD) values at 450 nm, measured using an enzyme-labeling instrument (Biotek, China).

### Plate cloning assay

Cells were taken, digested, and counted, and their density was adjusted to 1,000 cells/well. The 6-well plate was placed in a cell incubator. After 14 days, the cell colonies could be observed with the naked eye. One milliliter of phosphate-buffered saline (PBS) was used for cleaning. Then, 1 mL of 4% paraformaldehyde (Solarbio, China) was added to fix the cells for 30 min; after discarding the paraformaldehyde, the wells were washed twice with PBS. Then, 500 μL of 0.1% crystal violet (Solarbio, China) was added for 15 min. The crystal violet stain was discarded, and the wells were washed thrice with PBS. Finally, the plates were put into the oven to dry, observed, and photographed.

### Wound scratch assay

Cells were collected, digested, counted, and cultured in a 6-well plate (the bottom of the 6-well plate was marked with five equal-spacing horizontal lines in advance). When the cell growth reached 95% of the fusion degree, a vertical line was drawn on the cell surface using a 200-μL gun tip. Then, the supernatant was discarded, it was washed with PBS twice, and fresh 1640 serum-free medium was added. The cells were cultured for 24 h, 48 h, and 72 h. At the corresponding time point, the migration of the cells was observed using a microscope, and the images were captured.

### Invasion assay

Matrigel (ABW, China) was mixed with the serum-free medium at a ratio of 1:15 and added to the Transwell chamber (BIOFIL, China); the chamber was then placed in a 24-well plate and incubated in a refrigerator at 4°C overnight. The next day, the chamber was kept in a cell incubator at 37°C for 2 h, and the cells were resuspended with the serum-free medium. The suspension was planted on the upper part of the chamber, and 1640 medium with 30% FBS was added to the lower part of the chamber; the 24-well plate was cultured for 48 h. After 48 h, the cells were fixed with 4% paraformaldehyde for 30 min. Then, the chamber was stained with 0.1% crystal violet for 15 min. PBS was used to wash the top and bottom of the chamber, and it was allowed to dry after washing. The cells were observed under a microscope and photographed.

### Apoptosis assay

A total of 1 × 10^6^ cells were collected (cell supernatant was recovered), washed with precooled PBS, and centrifuged, and the supernatant was discarded. The cells were added with 500 μL of 1× binding buffer and then transferred to the corresponding flow tube (BD, US). Then, 10 μL of 7-AAD and 5 μL of Annexin V-APC (MultiSciences, China) were added to each tube, swirled gently, and incubated at room temperature in the dark for 5 min. The analysis was performed using a Canto high-speed analytical flow cytometer (BD, US).

### Cell cycle assay

A total of 1 × 10^6^ cells were collected, and 1 mL of PBS was added to the cells at room temperature. The cells were slowly added into precooled 3 mL anhydrous ethanol, quickly mixed, and placed in the refrigerator at −20°C overnight. The next day, the fixed cells were centrifuged, and the supernatant was discarded. Then, 3 mL of PBS was added to the cells at room temperature for 15 min to rehydrate the cells; the cells were centrifuged, and the supernatant was discarded. Next, 1 mL of DNA staining (MultiSciences, China) solution was added, mixed well, and transferred to a flow tube. The samples were then incubated at room temperature in the dark for 30 min, followed by performing the analysis using a Canto high-speed analytical flow cytometer.

### Immunofluorescence (IF)

Antibodies of anti-LC3 (1:100, CST) and a fluorescent secondary antibody (1:250, Proteintech) were purchased and used. The cells were cultured in 35 mm glass-substrate dishes (Biosharp, China) and washed with PBS when cell growth and fusion reached 95%. Cells were fixed with 4% paraformaldehyde on a shaker for 10 min, followed by washing with PBS on a shaker. Then, 0.2% Triton solution (Sigma, US) was added to the cells for 5 min, and the cells were again washed using PBS on a shaker. A 5% goat serum sealer (Solarbio, China) was added, and cells were incubated on a shaker for 1h, followed by another PBS wash. The corresponding antibodies were diluted with antibody diluent, followed by incubation of secondary antibodies in the dark. Cells were added with 10% DAPI (Solarbio, China) and stained away from light for 5 min. A two-photon laser confocal microscope (Carl Zeiss, German) was used to observe, analyze, and capture images.

### Drug sensitivity assay

The cell density was adjusted to 1 × 10^4^ cells/well. When the cell growth reached the fusion density of 80%, RA (0.62 nmol/L, 5 h) and CQ (300 μmol/L, 12 h) were applied to the corresponding cells, and then, Oxa was applied to the above cells for 24 h according to a certain concentration gradient. After 24 h, the liquid was discarded in the 96-well plate, and 100 μL of 10% CCK8 solution was added to each well; the cells were then incubated in an incubator at 37°C for 2 h. The OD value at 450 nm was detected using an enzyme-labeling instrument. Inhibition rate = (OD value of the blank well at 24 h −OD value of the experimental well at 24 h)/(OD value of the blank well at 24 h −OD value of the zero well at 24 h) ×100%. Among them, blank well contained cells and medium, zero well contained only medium, and experimental well contained cells, Oxa, and medium.

### Full transcriptome sequencing analysis

We commissioned Novogene (China) to perform full transcriptome sequencing on FEZF1-AS1 knocked down RKO cells (n = 3) and FEZF1-AS1 control RKO cells (n = 3). The process mainly included sample preparation, RNA sample detection, library construction, library inspection, sequencing, and bioinformatic analysis.

### Western blotting (WB)

Antibodies of anti-PI3K-P85α (1:1000, Proteintech), anti-AKT (1:1000, CST), anti-mTOR (1:1000, CST), anti-p-AKT (1:1000, CST), anti-p-mTOR (1:1000, CST), anti-LC3 (1:1000, CST), anti-p62 (1:1000, CST), and anti-GAPDH (1:50000, Proteintech) were purchased and used. RIPA lysate (Beyotime, China), phosphatase inhibitor (Beyotime, China), and PMSF protease inhibitor (Beyotime, China) were added into the cells, mixed evenly, and placed on ice for cracking for 30 min. Cell lysates were centrifuged in a high-speed refrigerated centrifuge at 12,000 rpm for 15 min at 4°C. Then, the supernatant was collected to measure the protein concentration using the BCA Protein Kit (Biosharp, China). The SDS-PAGE gel (Solarbio, China) was used for electrophoresis, and the NC membrane (PALL, US) was used for electrotransfer; the protein was transferred to the membrane. Then, 5% skim milk (Solarbio, China) was used for blocking for 1 h, followed by dilution of the corresponding antibodies with antibody diluent at the appropriate ratio and incubation. Finally, the NC membrane was exposed using a chemiluminescent gel image system (P&Q, China).

### Xenograft tumor models

Female BALB/c nude mice, aged 4 weeks, were acquired from Gempharmatech (China, Nanjing). Cells were resuspended with the appropriate serum-free medium and placed on ice for using. In the SPF animal room, 1 × 10^7^ cells in a volume of 200 μL were injected subcutaneously on the right backside of nude mice near the armpit, and any abnormalities were observed. On the second day, the mental state and activity of the nude mice were observed. After subcutaneous tumor was found in the nude mice, the length and width of the subcutaneous tumor were measured using a vernier caliper (DEGUQMNT, China) every 2 days, and the volume of the tumor was determined using the following formula: length × width^2^/2. On the 35th day, nude mice were treated intravenously with 3% pentobarbital sodium (Solarbio, China), and the tumors were removed, measured, and photographed. The fresh specimens were washed with PBS, cut into 1 × 1 cm tissues, and immediately placed in 4% formalin-fixing solution (Solarbio, China) for 24 h.

### Immunohistochemistry (IHC)

Antibodies of anti-PI3K-P85α (1:100, Proteintech), anti-AKT (1:100, CST), anti-mTOR (1:100, CST), anti-p-AKT (1:100, CST), anti-p-mTOR (1:100, CST), anti-LC3 (1:100, CST), and anti-p62 (1:100, CST) were purchased and used. The automatic dehydrator (Leica, German), paraffin embedding machine (Leica, German), and paraffin microtome (Leica, German) were used to dehydrate, embed, and slice the subcutaneous tumors, respectively. The slices were hydrated according to different ethanol concentration gradients, and citric acid tissue antigen repair solution (MXB, China) was used for tissue repair. Then, relevant reagents of the immunohistochemical kit (Servicebio, China) were used to treat with slices. The corresponding antibodies were diluted with an antibody diluent at a ratio of 1:100, followed by incubation. The slices were subjected to DAB (MXB, China) color development, followed by hematoxylin staining (Servicebio, China), bluing with differentiation solution, dehydration, and sealing with neutral gum (Solarbio, China). The sections were scanned using a panoramic histiocytic quantification system (TG, Austria). The Ipwin32 software system was used for staining positive analysis.

### Statistical analysis

IBM SPSS 23 was used for data statistics; GraphPad Prism8 and Origin96 were used for mapping. In the analysis of data results, the Student t test was used when the variance was homogeneous, whereas the Kruskal–Wallis test and Mann–Whitney U test were used when the variance was uneven. COX regression was used to analyze CC-related risk factors. Using the Kaplan–Meier method, we plotted the survival curve and compared it with the log-rank test. The correlation was examined using the Mann–Whitney U test and the Kruskal–Wallis test between the clinicopathological parameters of CC patients and FEZF1-AS1. P < 0.05 was considered statistically significant.

## Results

### FEZF1-AS1 expression was upregulated in CC tissues

**FIGURE 1:**
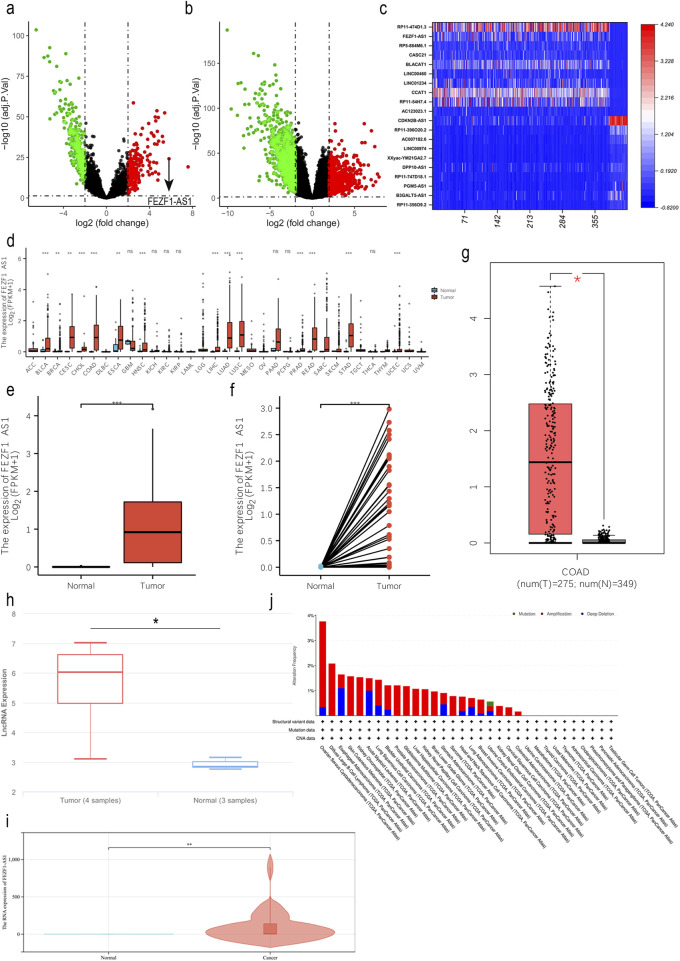
FEZF1-AS1 expression was upregulated in CC tissues. **(a** and **b)** The volcano maps of DElncRNAs and DEmRNAs in CC (|logFC| > 2, adj. P. Val <0.01) based on the TCGA dataset. Red represents the upregulated RNAs and green represents the downregulated RNAs. **(c)** In CC, heat map of DElncRNAs with expression levels in the top 10 and the bottom 10 based on the TCGA dataset. The X-axis represented 387 CC tissues and 38 normal colon tissues, and the Y-axis represents DElncRNAs. **(d)** In the TCGA database, the expression of FEZF1-AS1 in pan-cancer. **(e** and **f)** Expression of FEZF1-AS1 in unpaired or paired CC and normal colon tissues based on the TCGA dataset. **(g** and **h)** Expression of FEZF1-AS1 in CC and normal colon tissues based on GEPIA and lnCAR databases. **(i)** Expression of FEZF1-AS1 in 36 pairs of fresh CC and normal colon tissues. **(j)** In the cBioPortal database, FEZF1-AS1 showed an amplification mutation in CC. *, P < 0.05; **, P < 0.01; ***, P < 0.001; ns, no significance.

In the TCGA database, we obtained differentially expressed lncRNAs (DElncRNAs) and DEmRNAs between normal colon tissues and CC tissues through bioinformatic analysis. It indicated that there were 507 DElncRNAs (314 downregulated and 193 upregulated) and 1,732 DEmRNAs (684 upregulated and 1,048 downregulated, Figures 1a,b). The expression of FEZF1-AS1 in CC was very high (Figure 1C); we identified FEZF1-AS1 as the target molecule for our study. In NCBI, NONCODE, and UCSC databases, FEZF1-AS1 was in a low-expression pattern in normal colon tissues (Supplementary Figure S2). We analyzed CC datasets from the TCGA, GEPIA, and lnCAR databases and discovered an increased expression of FEZF1-AS1 mRNA in CC cells compared to normal colon tissues (Figures 1d–h). Additionally, through qRT-PCR, we detected 36 pairs of CC and normal colon tissues and found that the expression of FEZF1-AS1 was significantly higher in CC tissues than in normal colon tissues (Figure 1i). The cBioPortal database showed that FEZF1-AS1 exhibited an amplification mutation in CC.

### FEZF1-AS1 was enriched into several signaling pathways closely related to tumors, including the PI3K/AKT signaling pathway

**FIGURE 2:**
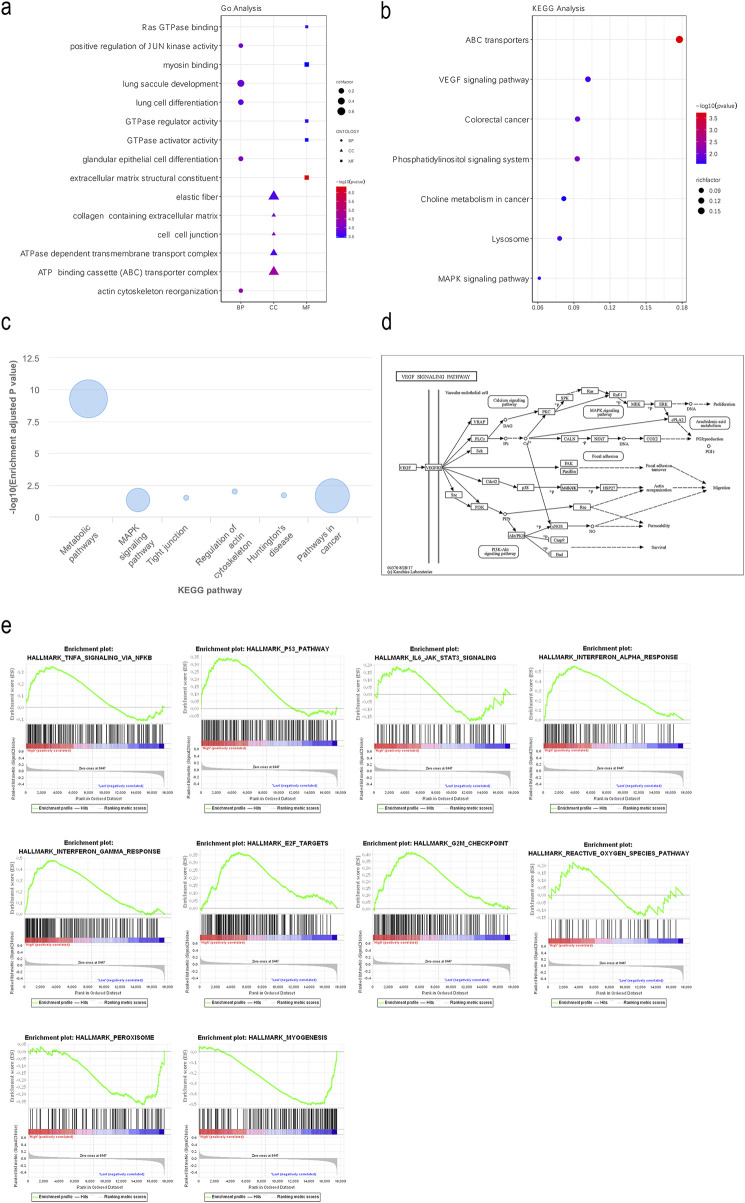
FEZF1-AS1 was enriched into several signaling pathways closely related to tumors, including the PI3K/AKT signaling pathway. **(a** and **b)** GO and KEGG functional enrichment analyses of FEZF1-AS1 and its co-expressed DEmRNAs based on the TCGA dataset. **(c)** KEGG functional enrichment analysis of FEZF1-AS1 in the lnCAR database. **(d)** VEGF activated the downstream PI3K/AKT signaling pathway in the KEGG database. **(e)** GSEA of FEZF1-AS1 and its co-expressed DEmRNAs based on the TCGA dataset.

In the TCGA and lnCAR databases, functional enrichment analyses of GO and KEGG showed that FEZF1-AS1 and its co-expressed DEmRNAs were enriched to the positive regulations of JUN kinase activity, the ATP binding cassette (ABC) transporter complex (Figure 2a), the VEGF signaling pathway, the colorectal cancer signaling pathway, the MAPK signaling pathway, ABC transports, lysosome (Figure 2b), the tumor-related signaling pathway, and tight junction (Figure 2c). The KEGG database showed that the VEGF signaling pathway initiated the subsequent PI3K/AKT signaling pathway (Figure 2d). We used GSEA enrichment analysis to further explore the potential function of FEZF1-AS1. The findings revealed that the gene set with high expression of FEZF1-AS1 in CC patients was enriched to the TNFα-NF-κB, P53, and IL-6-STAT3 signaling pathways and the reactive oxygen species (ROS) pathway (Figure 2e). All of these pathways were closely related to tumor development.

### FEZF1-AS1 had a high diagnostic value for CC and was correlated with clinicopathological parameters of CC patients

**FIGURE 3:**
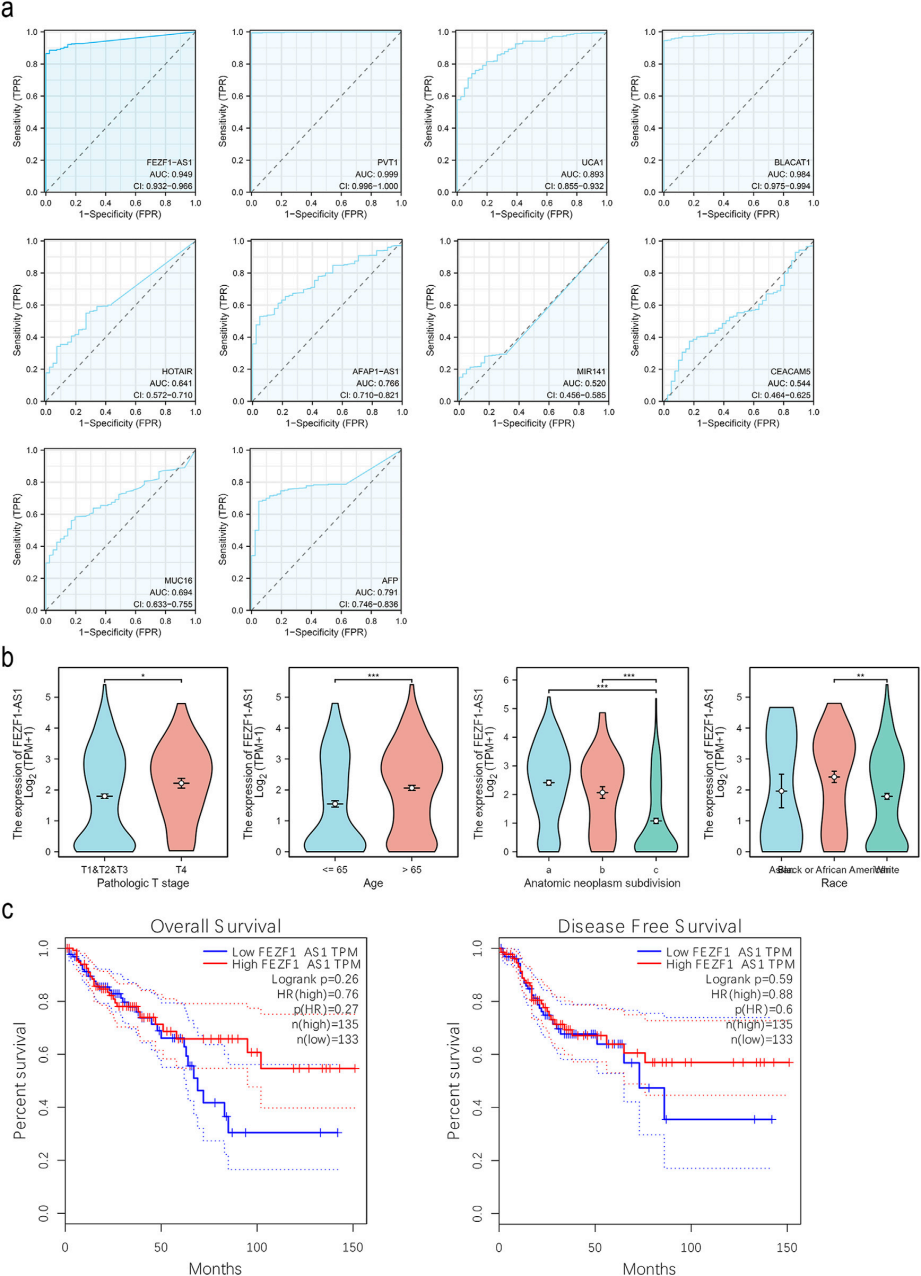
FEZF1-AS1 had a high diagnostic value for CC and was correlated with clinicopathological parameters of CC patients. **(a)** ROC curves of FEZF1-AS1, PVT1, UCA1, BLACAT1, HOTAIR, AFAP1-AS1, MIR141, CEACAM5 (CEA), MUC16 (CA-125), and AFP in CC based on the TCGA dataset. **(b)** Expression of FEZF1-AS1 in patients with CC at different T stages, ages, tumorigenic sites (a, right colon; b, transverse colon; c, left colon), and race based on the TCGA dataset. **(c)** Kaplan–Meier evaluation of OS and DFS of cases from the GEPIA database. *, P < 0.05; **, P < 0.01; ***, P < 0.001.

ROC analysis indicated that the AUC value of FEZF1-AS1 in CC was 0.949, whereas the AUC values of other genes with reported diagnostic values in other studies ranged from 0.520 to 0.999, indicating that FEZF1-AS1 had a high diagnostic value for CC (Figure 3a). An analysis was conducted on the correlation between the clinicopathological characteristics of CC patients and FEZF1-AS1 in the TCGA database. It was found that the expression of FEZF1-AS1 in CC patients was different among different clinical parameter groups, such as pathologic T stage (P < 0.05), tumorigenic sites (P < 0.001), age (P < 0.001), and race (P < 0.01) (Figure 3b). Based on the current sample size, survival analysis in the GEPIA database showed that FEZF1-AS1 had no effect on overall survival (OS) and disease-free survival (DFS) in 268 patients with CC (Figure 3c). The COX regression analysis revealed a correlation among the age, pathologic T stage, N stage, clinical stage, and lymphatic invasion in CC patients and their OS, and could be used as independent prognostic factors for CC (Table 1).

**TABLE 1:** COX regression analysis.

Characteristic	Total (N)	Univariate analysis		Multivariate analysis	
		HR (95% CI)	P	HR (95% CI)	P
Age (years)	477				
≤65	194	Reference		Reference	
>65	283	1.610 (1.052–2.463)	0.028	3.581 (1.617–7.930)	0.002
Gender	477				
Female	226	Reference			
Male	251	1.101 (0.746–1.625)	0.627		
Race	306				
Asian	11	Reference			
Black or African American	63	0.927 (0.208–4.133)	0.921		
White	232	0.810 (0.196–3.346)	0.771		
Pathologic T stage	476				
T1, T2, and T3	416	Reference		Reference	
T4	60	3.152 (1.941–5.118)	<0.001	3.330 (1.542–7.192)	0.002
Pathologic N stage	477				
N0	283	Reference		Reference	
N1 and N2	194	2.592 (1.743–3.855)	<0.001	0.175 (0.036–0.859)	0.032
Pathologic M stage	414				
M0	348	Reference		Reference	
M1	66	4.193 (2.683–6.554)	<0.001	1.393 (0.619–3.136)	0.423
Clinical stage	466				
Stages I and II	267	Reference		Reference	
Stages III and IV	199	2.947 (1.942–4.471)	<0.001	16.822 (2.689–105.218)	0.003
Lymphatic invasion	433				
No	265	Reference		Reference	
Yes	168	2.450 (1.614–3.720)	<0.001	2.553 (1.132–5.762)	0.024
CEA	302				
≤5	195	Reference		Reference	
>5	107	3.128 (1.788–5.471)	<0.001	1.613 (0.785–3.315)	0.193
FEZF1-AS1	477				
Low	238	Reference			
High	239	0.985 (0.668–1.452)	0.938		

### FEZF1-AS1 promoted the progression of CC

**FIGURE 4:**
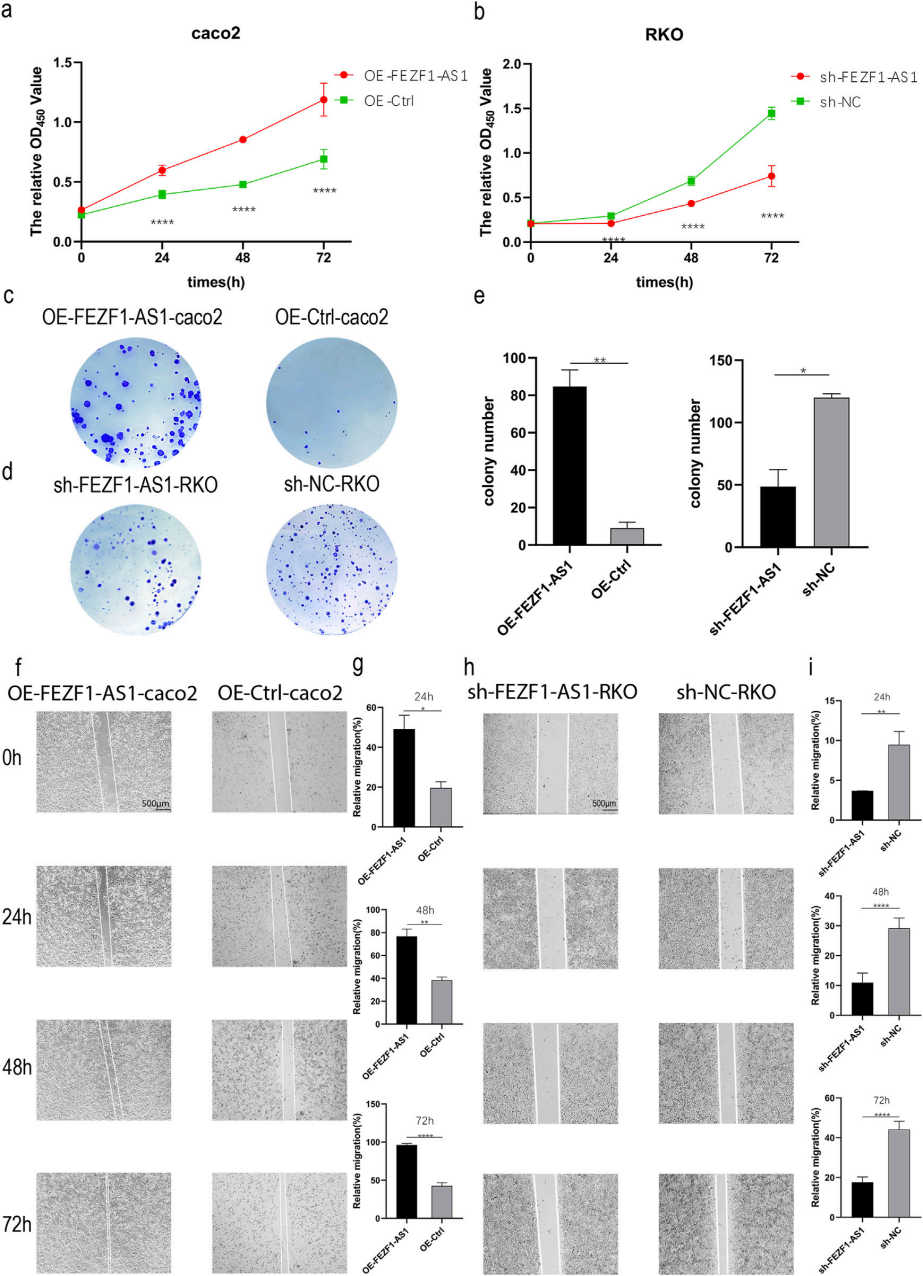
FEZF1-AS1 promoted the proliferation and migration of CC cells. **(a** and **b)** Growth curves of the cells at 24 h, 48 h, and 72 h were detected using the CCK8 assay. Red represents the OE-FEZF1-AS1-Caco2 or sh-FEZF1-AS1-RKO cell, and green represents the OE-Ctrl-Caco2 or sh-NC-RKO cell. **(c** and **d)** Colony formation of the cells at 14 days. Blue dots on the panel represent cell colonies. **(e)** Statistical analysis of colony formation. **(f** and **h)** The migration of cells at 0 h, 24 h, 48 h, and 72 h was detected using the scratch assay. **(g** and **i)** Statistical analysis of scratch assay. *, P < 0.05; **, P < 0.01; ****, P < 0.0001.

**FIGURE 5:**
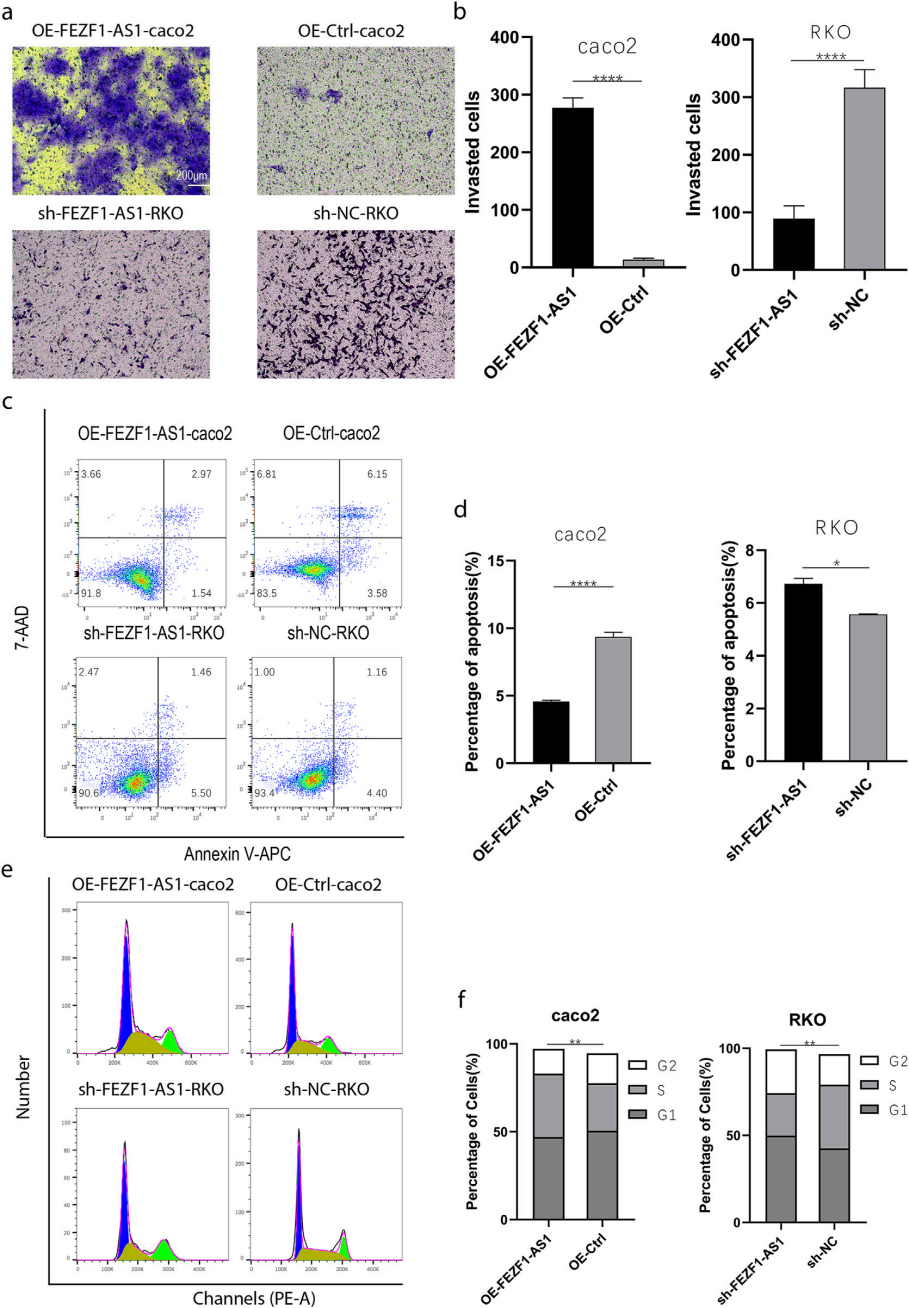
FEZF1-AS1 promoted the invasion, survival, and proliferation of CC cells. **(a)** The invasion of cells at 48 h was detected using the Transwell assay. **(b)** Statistical analysis of invasion. **(c)** The apoptosis of cells was detected through flow cytometry. **(d)** Statistical analysis of apoptosis. **(e)** Cell cycle was detected through flow cytometry. **(f)** Statistical analysis of cell cycle. *, P < 0.05; **, P < 0.01; ****, P < 0.0001.

**FIGURE 6:**
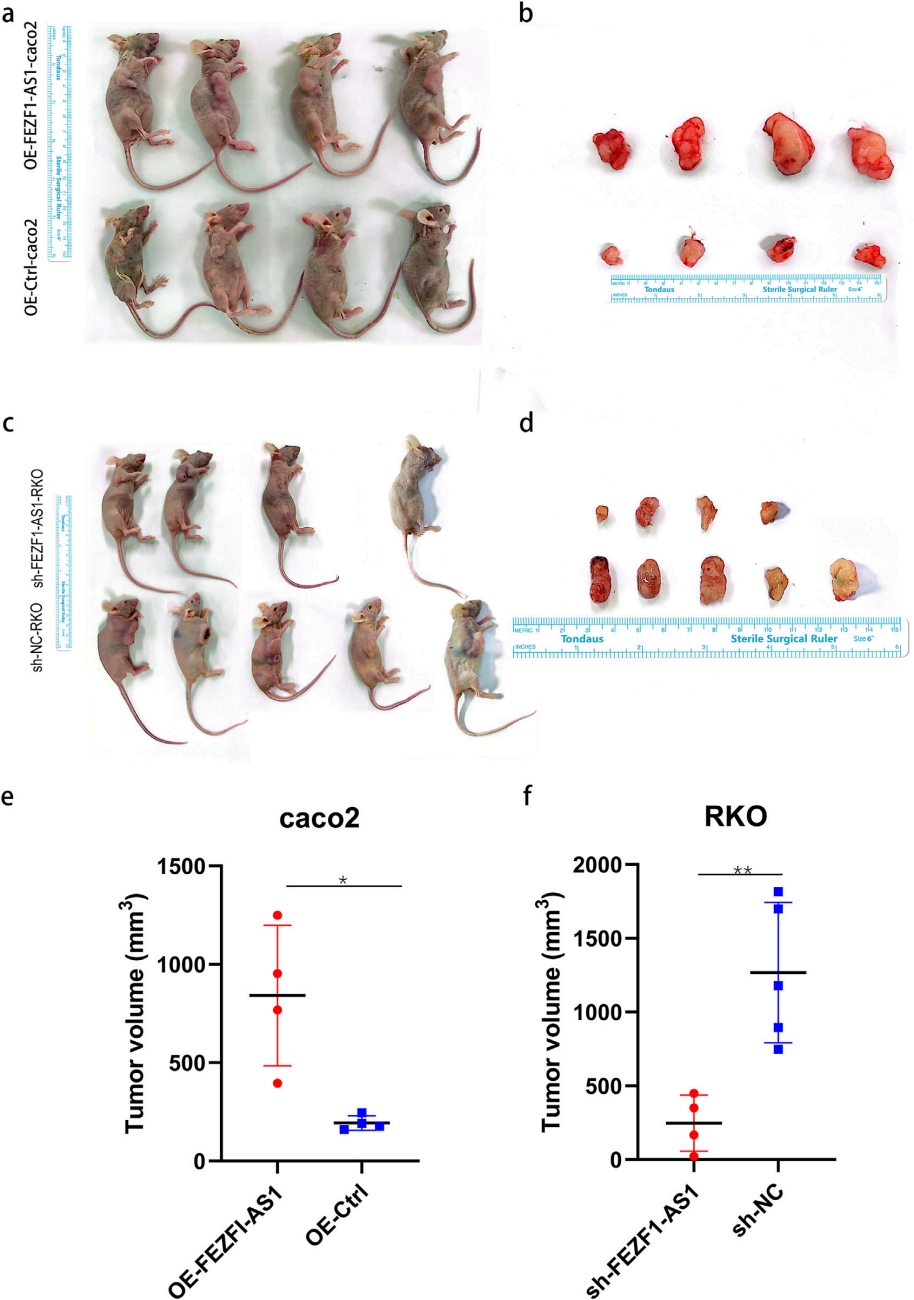
FEZF1-AS1 promoted the tumor formation of CC cells in nude mice. **(a** and **c)** Subcutaneous tumor formation of cells in nude mice. **(b** and **d)** Subcutaneous tumor tissue specimens. **(e** and **f)** Statistical analysis of the volumes of tissue specimens. *, P < 0.05; **, P < 0.01.

qRT-PCR was used to identify the expression of FEZF1-AS1 in CC cells. The results showed that compared with NCM460 cell, the expression of FEZF1-AS1 was relatively higher in Caco2 and RKO cells (Supplementary Figure S3A). Therefore, we selected RKO cell for lentivirus FEZF1-AS1-knocked down transfection and Caco2 cell for lentivirus FEZF1-AS1-overexpressed transfection, which were named sh-FEZF1-AS1-RKO and sh-NC-RKO cells, and OE-FEZF1-AS1-Caco2 and OE-Ctrl-Caco2 cells, respectively. Lentivirus fluorescence transfection efficiency reached 100% (Supplementary Figure S3B). Compared with the OE-Ctrl-Caco2 cell, the expression of FEZF1-AS1 in the OE-FEZF1-AS1-Caco2 cell was notably increased, and the overexpressed efficiency could reach 2,600 times (Supplementary Figure S3C); the proliferation, migration, invasion, and cycle (S-phase cell proportion) of the OE-FEZF1-AS1-Caco2 cell were significantly increased, and apoptosis was significantly decreased (Figures 4, 5). Compared with that in the sh-NC-RKO cell, the expression of FEZF1-AS1 in the sh-FEZF1-AS1-RKO cell was decreased significantly, and the knocked down efficiency reached 85% (Supplementary Figure S3D); the proliferation, migration, invasion, and cycle (S-phase ratio) of sh-FEZF1-AS1-RKO were significantly reduced, and apoptosis was significantly increased (Figures 4, 5). The results of the tumor-bearing mouse experiment showed that the volumes of tumor formation in nude mice implanted with Caco2 cells overexpressing FEZF1-AS1 were significantly larger than those in control mice (Figures 6a,b). The volumes of tumor formation in nude mice implanted with RKO cells knocking down FEZF1-AS1 were significantly smaller than those in control mice (Figures 6c,d). All abovementioned results validated that FEZF1-AS1 facilitates the progression of CC.

### FEZF1-AS1 triggered autophagy in CC cells

CQ elevated the lysosome’s pH level, leading to a decrease in the lysosome’s acid hydrolase activity, thus impeding the degradation process of “autophagy lysosome.” Therefore, LC3 located on the autophagosome and autophagy lysosome membranes was delayed in degradation, leading to the accumulation of the LC3 protein and inhibiting the occurrence of autophagy. RA, as an inhibitor of mTOR, could activate autophagy.

**FIGURE 7:**
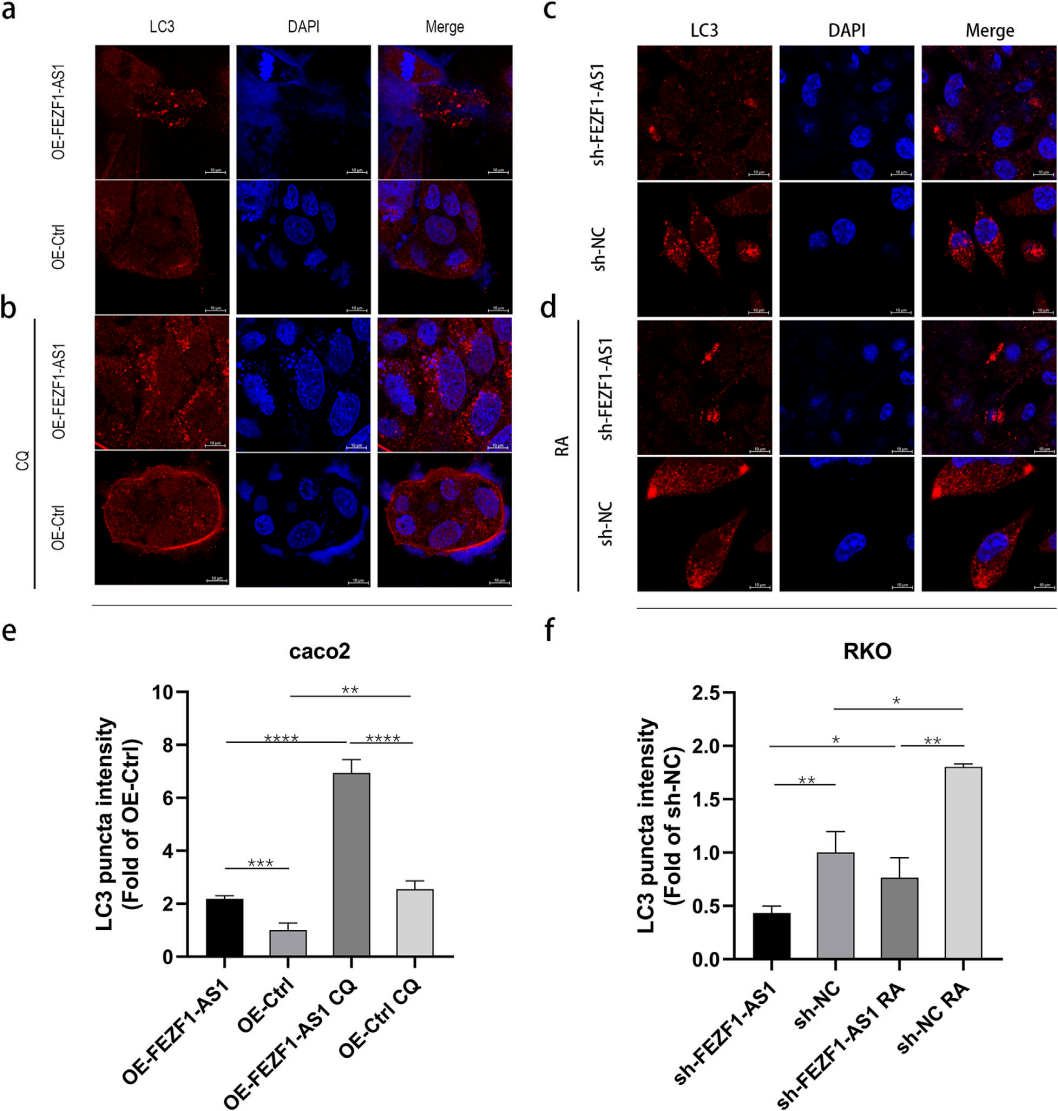
FEZF1-AS1 triggered autophagy in Caco2 and RKO cells. **(a** and **b)** LC3 expression in OE-FEZF1-AS1-Caco2 and OE-Ctrl-Caco2 cells with or without 300 μmol/L CQ for 12 h was detected using the IF assay. Brightest red dot clusters in the image represent the LC3 proteins. **(c** and **d)** LC3 expression in sh-FEZF1-AS1-RKO and sh-NC-RKO cells with or without 0.62 nmol/L RA for 5 h was detected using the IF assay. Brightest red dot clusters in the image represent the LC3 proteins. **(e** and **f)** Statistical analysis of LC3 expression. *, P < 0.05; **, P < 0.01; ***, P < 0.001; ****, P < 0.0001.

To confirm the influence of FEZF1-AS1 on autophagy, we used IF assay to detect a classical autophagy marker, LC3. The results showed that compared with that in OE-FEZF1-AS1-Caco2 and OE-Ctrl-Caco2 cells, LC3 expression was increased in both OE-FEZF1-AS1-CQ-Caco2 and OE-Ctrl-CQ-Caco2 cells. Among them, LC3 expression in the OE-FEZF1-AS1-Caco2 cell was significantly higher than that in the OE-Ctrl-Caco2 cell with or without CQ. Compared with that in sh-FEZF1-AS1-RKO and sh-NC-RKO cells, LC3 expression was increased in sh-FEZF1-AS1-RA-RKO and sh-NC-RA-RKO cells. The expression of LC3 in the sh-FEZF1-AS1-RKO cell was significantly less than that in the sh-NC-RKO cell with or without RA (Figure 7).

### FEZF1-AS1 promoted the progression of CC and reduced the sensitivity to oxa through autophagy

**FIGURE 8:**
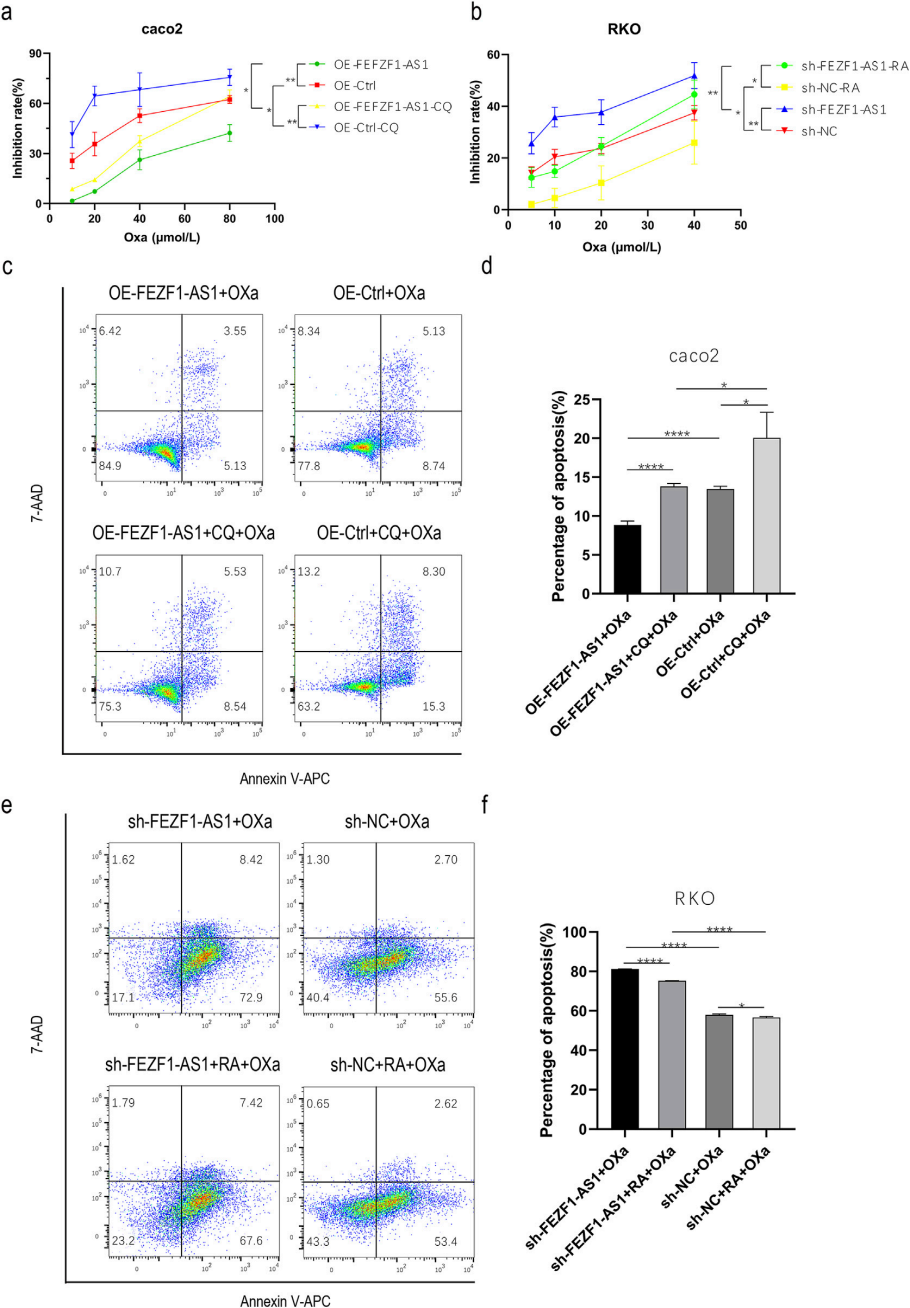
FEZF1-AS1 promoted the progression of CC and reduced the sensitivity to Oxa through autophagy. **(a)** Growth inhibition curves of cells, regardless of whether Oxa was mixed with CQ or not. **(b)** Growth inhibition curves of cells with treatments of Oxa combined with RA or without it. **(c)** Apoptosis of cells, regardless of whether Oxa was mixed with CQ or not. **(d)** Statistical analysis of apoptosis. **(e)** Apoptosis of cells with treatments of Oxa combined with RA or without it. **(f)** Analysis of apoptosis statistics. *, P < 0.05; **, P < 0.01; ****, P < 0.0001.

We first treated OE-FEZF1-AS1-Caco2 and OE-Ctrl-Caco2 cells with or without 300 μmol/L CQ for 12 h, respectively. Then, OE-FEZF1-AS1-Caco2 and OE-Ctrl-Caco2 cells were treated with Oxa at a certain concentration gradient (10, 20, 40, and 80 μmol/L) for 24 h. The results showed that the growth inhibition rate of OE-FEZF1-AS1-Caco2 cells was significantly lower than that of OE-Ctrl-Caco2 cells, and the sensitivity to Oxa was lower. The growth inhibition rate of OE-FEZF1-AS1-CQ-Caco2 cells was significantly lower than that of OE-Ctrl-CQ-Caco2 cells, and the sensitivity to Oxa was lower. The growth inhibition rate of OE-FEZF1-AS1-Caco2 cells was significantly lower than that of OE-FEZF1-AS1-CQ-Caco2 cells, and the sensitivity to Oxa was lower. OE-Ctrl-Caco2 cells had a significantly lower growth inhibition rate than OE-Ctrl-CQ-Caco2 cells and were less sensitive to Oxa (Figure 8a).

We treated sh-FEZF1-AS1-RKO and sh-NC-RKO cells with or without 0.62 nmol/L RA for 5 h, respectively. Then, sh-FEZF1-AS1-RKO and sh-NC-RKO cells were treated with Oxa at a certain concentration gradient (5, 10, 20, and 40 μmol/L) for 24 h. The results showed that the growth inhibition rate of sh-FEZF1-AS1-RKO cells was significantly higher and more sensitive to Oxa than that of sh-NC-RKO cells. The growth inhibition rate of sh-FEZF1-AS1-RA-RKO cells was significantly higher and more sensitive to Oxa than that of sh-NC-RA-RKO cells. The growth inhibition rate of sh-NC-RKO cells was significantly higher and more sensitive to Oxa than that of sh-NC-RA-RKO cells. The growth inhibition rate of sh-FEZF1-AS1-RKO cells was significantly higher and more sensitive to Oxa than that of sh-FEZF1-AS1-RA-RKO cells (Figure 8b).

We first treated OE-FEZF1-AS1-Caco2 and OE-Ctrl-Caco2 cells with or without 300 μmol/L CQ for 12 h, respectively. Then, 50 μmol/L Oxa was added to OE-FEZF1-AS1-Caco2 and OE-Ctrl-Caco2 cells for 12 h, and apoptosis was determined using flow cytometry. We found that the apoptosis ratio of OE-FEZF1-AS1-Caco2 cells was significantly lower than that of the OE-Ctrl-Caco2 cell, and the sensitivity to Oxa was lower. The apoptosis ratio of OE-FEZF1-AS1-CQ-Caco2 cells was significantly lower than that of OE-Ctrl-CQ-Caco2 cells, and the sensitivity to Oxa was lower. The apoptosis ratio of OE-FEZF1-AS1-Caco2 cells was significantly lower than that of OE-FEZF1-AS1-CQ-Caco2 cells, and the sensitivity to Oxa was lower. OE-Ctrl-Caco2 cells had a significantly lower percentage of apoptosis than OE-Ctrl-CQ-Caco2 cells and were less sensitive to Oxa (Figure 8c).

We treated sh-FEZF1-AS1-RKO and sh-NC-RKO cells with or without RA at 0.62 nmol/L for 5 h and then added 20 μmol/L Oxa to sh-FEZF1-AS1-RKO and sh-NC-RKO cells for 24 h. We found that compared with that of sh-NC-RKO cells, the apoptosis ratio of sh-FEZF1-AS1-RKO cells was significantly increased, and the sensitivity to Oxa was higher. Compared with that of sh-NC-RA-RKO cells, the apoptosis ratio of sh-FEZF1-AS1-RA-RKO cells was significantly higher, and the sensitivity to Oxa was higher. Compared with that of sh-NC-RA-RKO cells, the apoptosis ratio of sh-NC-RKO cells was significantly higher, and the sensitivity to Oxa was higher. sh-FEZF1-AS1-RKO cells had a significantly higher percentage of apoptosis and a higher sensitivity to Oxa than sh-FEZF1-AS1-RA- RKO cells (Figure 8e). These results all indicated that FEZF1-AS1 promoted the progression of CC and reduced the sensitivity to Oxa through autophagy.

### Through RNA-seq, FEZF1-AS1 could facilitate CC development through the modulation of the PI3K/AKT signaling pathway, facilitating autophagy

**FIGURE 9:**
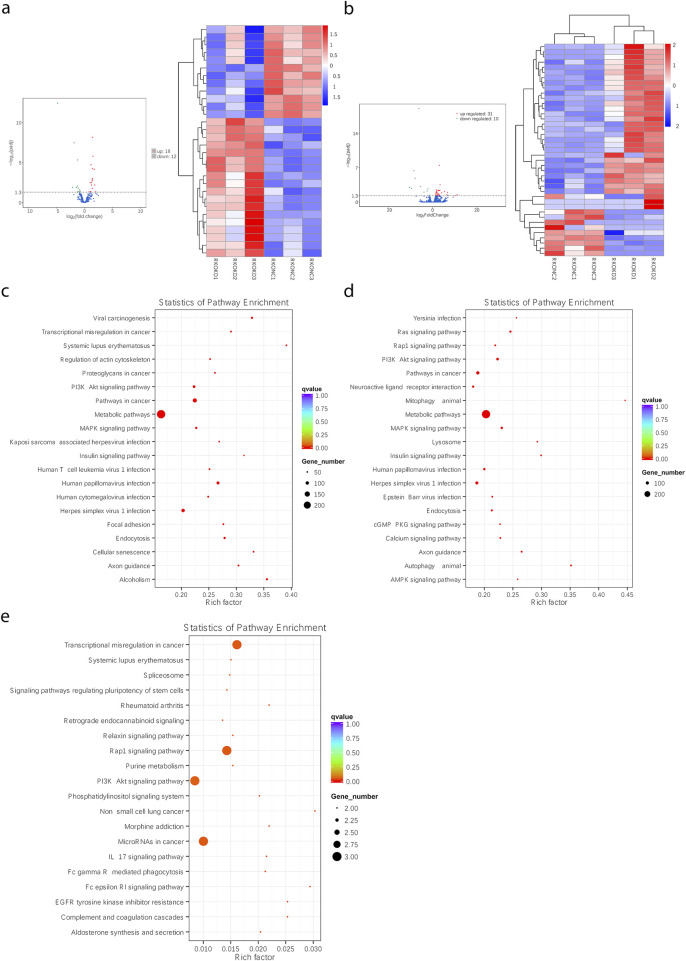
Through RNA-seq, FEZF1-AS1 could facilitate CC development through the modulation of the PI3K/AKT signaling pathway, facilitating autophagy. **(a** and **b)** The heat maps of DEmiRNAs and DEmRNAs between sh-FEZF1-AS1-RKO cells and sh-NC-RKO cells. **(c)** KEGG functional enrichment analysis of FEZF1-AS1 and its co-expressed gene set in sh-FEZF1-AS1-RKO and sh-NC-RKO cells. **(d)** KEGG functional enrichment analysis of the DEmiRNA and its co-expressed gene set in sh-FEZF1-AS1-RKO and sh-NC-RKO cells. **(e)** KEGG functional enrichment analysis of the DEmRNA and its co-expressed gene set in sh-FEZF1-AS1-RKO and sh-NC-RKO cells.

sh-FEZF1-AS1-RKO and sh-NC-RKO cells were analyzed through RNA-seq, and DEmiRNAs and DEmRNAs were obtained. Among them, compared with sh-NC-RKO cells, 18 upregulated DEmiRNA, 12 downregulated DEmiRNA, 31 upregulated DEmRNA, and 10 downregulated DEmRNA were found in sh-FEZF1-AS1-RKO cells (Figures 9a,b). The obtained differentially expressed genes were enriched into the PI3K/AKT signaling pathway, lysosome, autophagy, extracellular matrix, and other tumor-related pathways (Figures 9c–e; Supplementary Figure S4). We suggested that FEZF1-AS1 facilitated the development of CC by regulating the PI3K/AKT signaling pathway to mediate autophagy.

### FEZF1-AS1 suppressed the PI3K/AKT/mTOR signaling pathway to trigger autophagy and promote the development of CC

According to the results of functional enrichment analysis through RNA-seq, it was suggested that FEZF1-AS1 might promote CC development through the regulation of autophagy mediated by the PI3K/AKT/mTOR signaling pathway. Therefore, we detected the expression of key proteins in this pathway using WB technology, which was rescued by CQ and RA.

**FIGURE 10:**
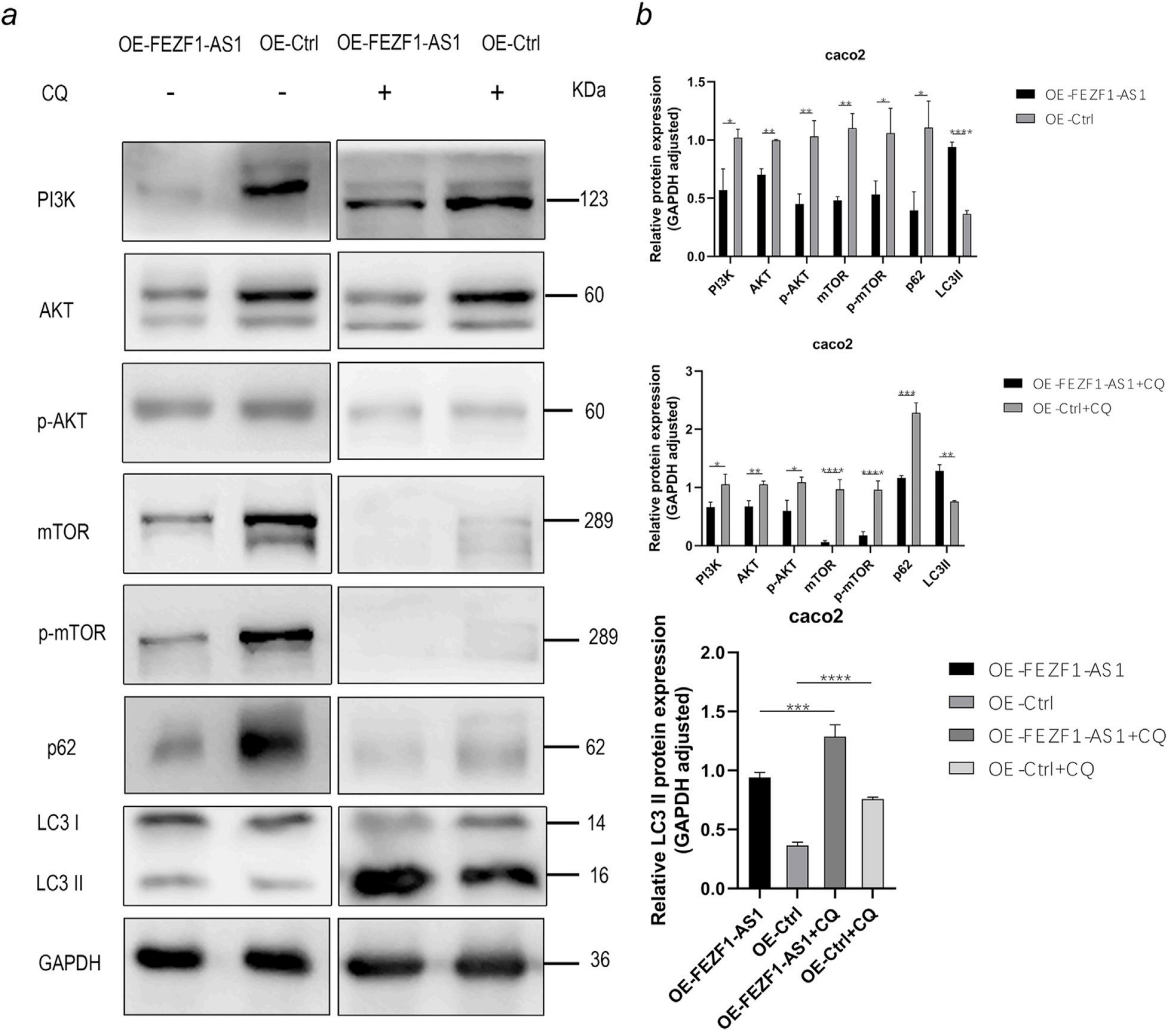
FEZF1-AS1 inhibited the PI3K/AKT/mTOR signaling pathway, thereby triggering autophagy and facilitating Caco2 development. **(a)** WB analysis of the PI3K/AKT/mTOR signaling pathway and autophagy marker proteins in OE-FEZF1-AS1-Caco2 and OE-Ctrl-Caco2 cells with or without CQ treatment. GAPDH served as an internal loading control. **(b)** Statistical analysis of the protein expressions. *, P < 0.05; **, P < 0.01; ***, P < 0.001; ****, P < 0.0001.

The results of WB showed that compared with those in OE-Ctrl-Caco2 cells, there was a reduction in the protein levels of PI3K, AKT, p-AKT, mTOR, p-mTOR, and p62 in OE-FEZF1-AS1-Caco2 cells, whereas LC3 II expression was increased. After a 12-h exposure to 300 μmol/L CQ, compared with those in OE-Ctrl-CQ-Caco2 cells, there was a reduction in the protein levels of PI3K, AKT, p-AKT, mTOR, p-mTOR, and p62 in OE-FEZF1-AS1-CQ-Caco2 cells, whereas LC3 II expression was increased. The protein level of LC3 II in OE-FEZF1-AS1-CQ-Caco2 cells was significantly higher than that of LC3 II in OE-FEZF1-AS1-Caco2 cells. The protein level of LC3 II in OE-Ctrl-CQ-Caco2 cells was significantly higher than that of LC3 II in OE-Ctrl-Caco2 cells (Figure 10a).

**FIGURE 11:**
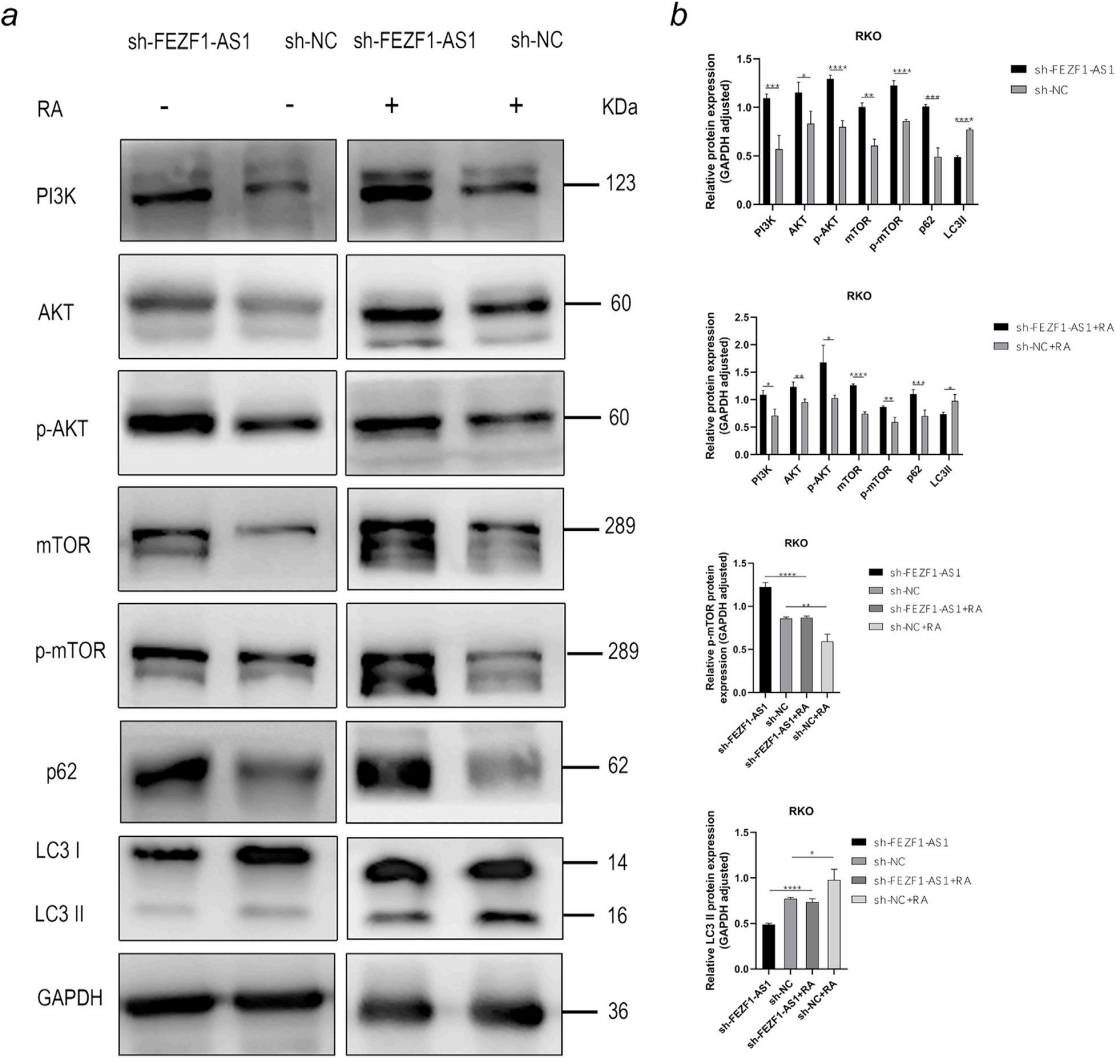
FEZF1-AS1 inhibited the PI3K/AKT/mTOR signaling pathway, thereby triggering autophagy and facilitating RKO development. **(a)** WB analysis of the PI3K/AKT/mTOR signaling pathway and autophagy marker proteins in sh-FEZF1-AS1-RKO and sh-NC-RKO cells with or without RA treatment. GAPDH served as an internal loading control. **(b)** Statistical analysis of the protein expressions. *, P < 0.05;**, P < 0.01; ***, P < 0.001; ****, P < 0.0001.

On the contrary, compared with those in sh-NC-RKO cells, there was an elevation in the protein levels of PI3K, AKT, p-AKT, mTOR, p-mTOR, and p62 in sh-FEZF1-AS1-RKO cells, whereas LC3 II expression was decreased. After 5 h treatment with 0.62 nmol/L RA, compared with those in sh-NC-RA-RKO cells, there was an elevation in the protein levels of PI3K, AKT, p-AKT, mTOR, p-mTOR, and p62 in sh-FEZF1-AS1-RA-RKO cells, whereas LC3 II expression was decreased. Compared with protein levels in sh-FEZF1-AS1-RKO cells, the protein level of p-mTOR was significantly reduced, and LC3 II was significantly increased in sh-FEZF1-AS1-RA-RKO cells. Compared with protein levels in sh-NC-RKO cells, the protein level of p-mTOR was significantly reduced, and LC3 II was significantly increased in sh-NC-RA-RKO cells (Figure 11a).

**FIGURE 12:**
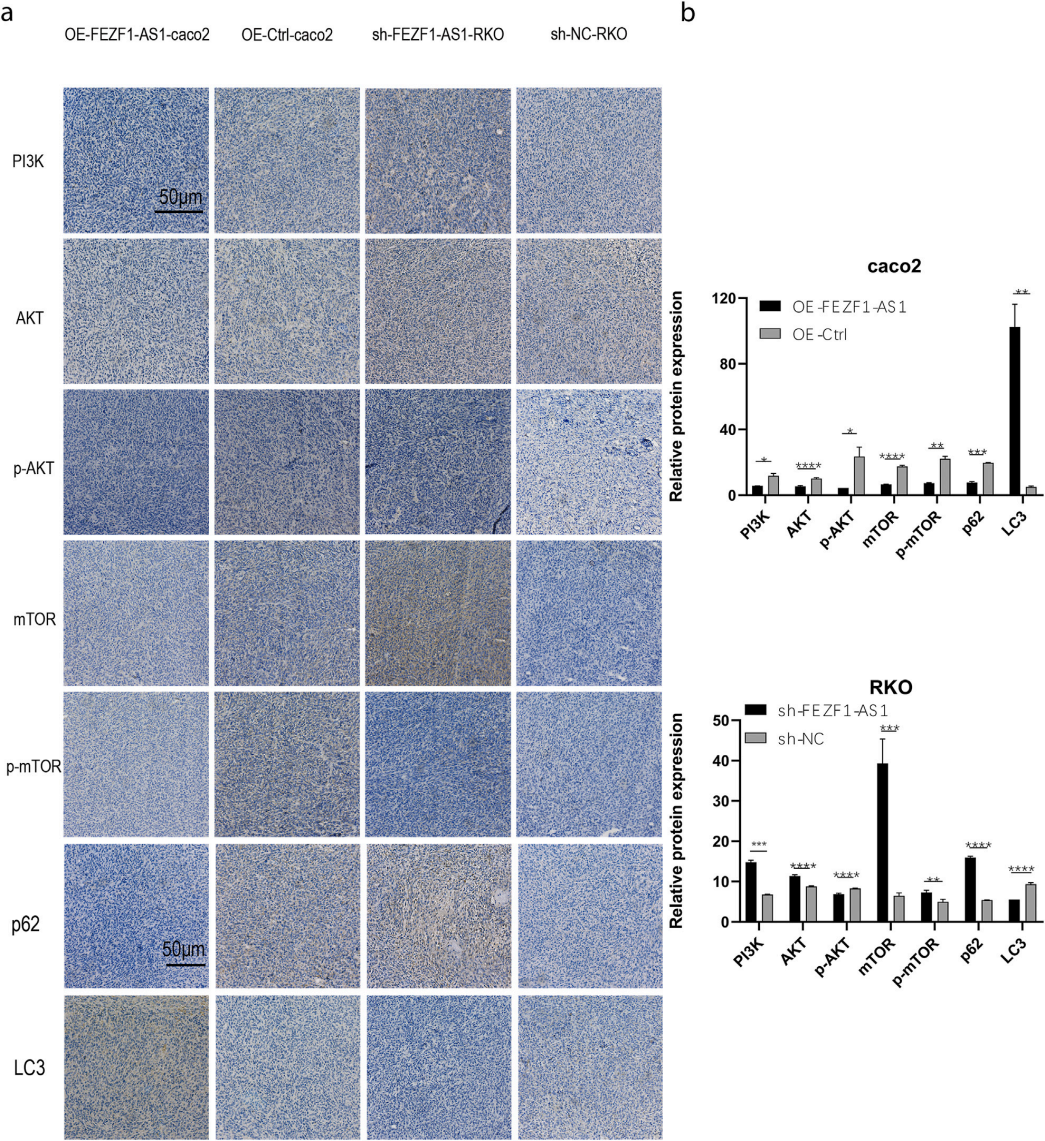
FEZF1-AS1 inhibited the PI3K/AKT/mTOR signaling pathway, thereby triggering autophagy and facilitating CC development. **(a)** IHC staining of key proteins of the PI3K/AKT/mTOR pathway and autophagy marker proteins in tumor-forming tissues of nude mice. Blue stain represents the nucleus and yellow stain represents the target protein. **(b)** Statistical analysis of the protein expressions. *, P < 0.05; **, P < 0.01; ***, P < 0.001; ****, P < 0.0001.

**FIGURE 13:**
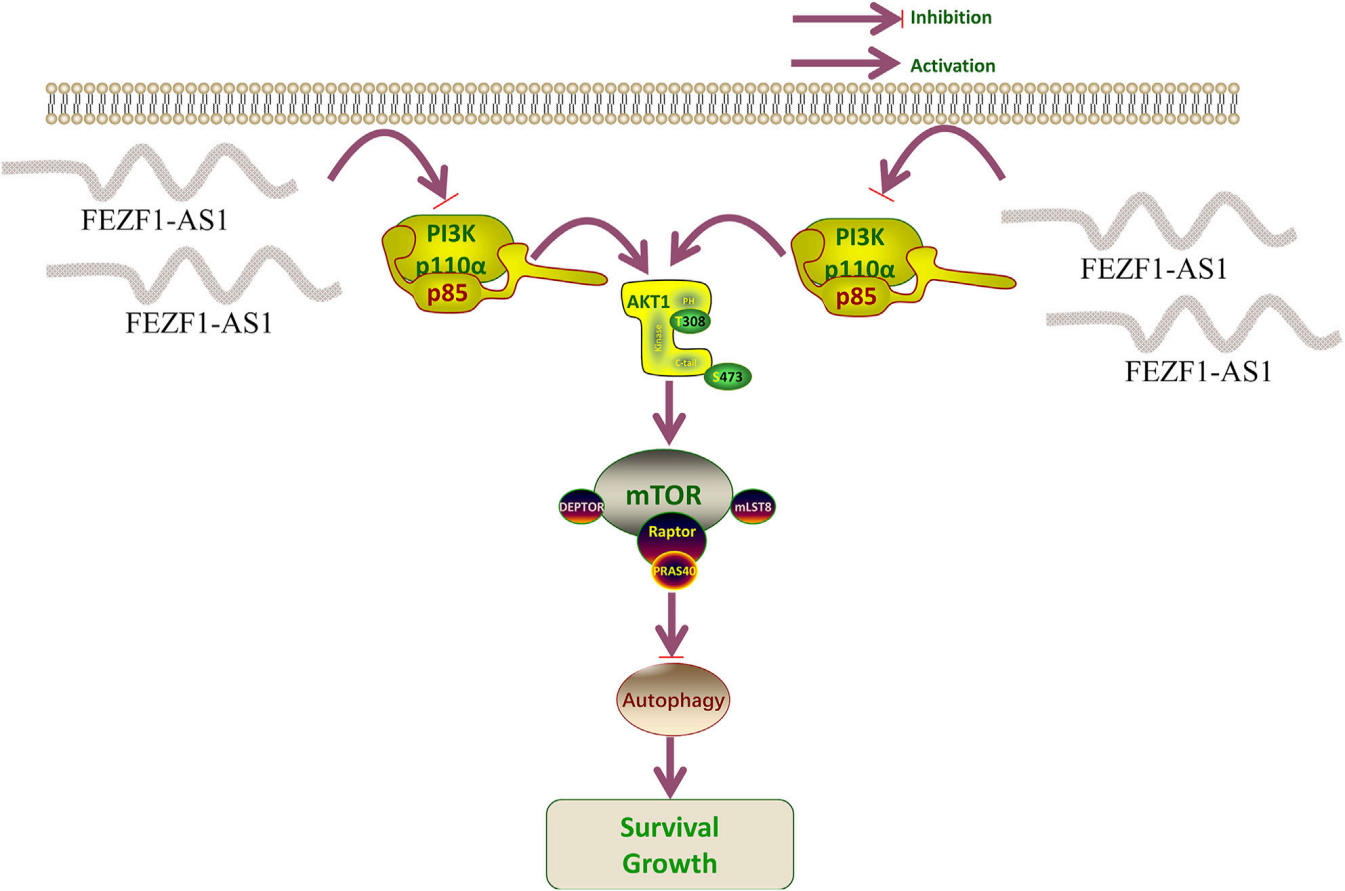
Manuscript flowchart.

The tumor tissues of nude mice were stained using IHC. The findings revealed that compared with those in the OE-Ctrl-Caco2 cell group, there was a reduction in the protein levels of PI3K, AKT, p-AKT, mTOR, p-mTOR, and p62 in the OE-FEZF1-AS1-Caco2 cell group, whereas the expression of LC3 was increased. On the contrary, compared with those in the sh-NC-RKO cell group, there was an elevation in the protein levels of PI3K, AKT, p-AKT, mTOR, p-mTOR, and p62 in the sh-FEZF1-AS1-RKO cell group, whereas LC3 expression was decreased (Figure 12). We believed that FEZF1-AS1 inhibited the PI3K/AKT/mTOR signaling pathway to trigger autophagy and promote the development of CC. The manuscript flowchart is shown in Figure 13.

## Discussion

At present, Oxa and capecitabine remain the first line of chemotherapy for CC (André et al., 2004). The rapid progression of CC, accompanied by acquired chemoresistance, recurrence, and metastasis, makes the prognosis of CC patients poor (Holohan et al., 2013). A study has shown that the activation of autophagy is one of the important reasons for promoting CC progression and chemoresistance (Wang et al., 2021), but the specific mechanism is still unclear.

Through bioinformatic analysis, many lncRNAs that are highly expressed in CC were discovered in our research, such as RP11-474D1.3, CASC21, BLACAT1, LINC00460, LINC01234, CCAT1, and RP11-54H7.4. Studies have shown that CDK6 is involved in the transformation of the G1 to S cell cycle, and the upregulation of CDK6 is related to the occurrence and development of multiple tumors (Tigan et al., 2016; Tadesse et al., 2015). In Gong’s study, CASC21 is highly expressed in CC. It promotes the expression of CDK6 by sponging miR-539-5p, thereby facilitating the cell cycle transition of CC cells (Gong et al., 2020). BLACAT1 inhibits the trimethylation of H3K27 on the CDKN1C gene by blocking the recruitment of EZH2, thereby promoting the expression of CDKN1C and inhibiting the expression of CCNE, and thus suppressing the biological processes of pancreatic cancer cells (Zhou et al., 2020). Based on the abovementioned research works, we believe that the lncRNA is involved in epigenetic regulation, acts as a competitive endogenous RNA, regulates protein stability and genomic localization indication function, participates in multiple tumor-related signaling pathways, and serves as a novel biomarker of prognosis, treatment, and other roles. The lncRNA FEZF1-AS1 is a new tumor gene recently discovered (Ye et al., 2018). Studies indicate a significant presence of FEZF1-AS1 in cases of liver cancer, gastric cancer, and epithelial ovarian cancer. Higher FEZF1-AS1 expression is linked to clinicopathological factors, such as lymph node metastasis, higher pathologic T stage, invasion, and distant metastasis, leading to lower survival rates in individuals with these tumors. It is also expected to be used as an important diagnostic and prognostic indicator for cancer patients (Sun et al., 2020; Wang et al., 2018; Liu et al., 2017; Wu et al., 2017). In our study, we find that FEZF1-AS1 is highly expressed in CC. The expression of FEZF1-AS1 in CC patients with the pathologic T4 stage is higher than that of FEZF1-AS1 in CC patients in T1–T3 stages, suggesting that FEZF1-AS1 may be related to the development of CC. There is an amplified mutation of FEZF1-AS1 in CC based on the cBioPortal database, indicating that the high expression of FEZF1-AS1 in CC is probably caused by the amplification mutation of FEZF1-AS1 at the gene level. It is suggested that FEZF1-AS1 may be an oncogenic gene of CC. It has shown that genetic mutations are present in many tumors. For example, driver mutations of RAS genes (KRAS type, NRAS, and HRAS) lead to infinite proliferation and enhanced survival of tumor cells (Karnoub and Weinberg, 2008). He et al. (2022) showed that the amplification and mutation of the HOXA5 gene are associated with glioma histology and it is a biomarker of independent prognosis of glioma. It is well known that CEA, CA-125, and AFP are the most common biological markers of CC. Multiple studies have reported that PVT1, UCA1, BLACAT1, HOTAIR, and AFAP1-AS1 are potential biomarkers for the diagnosis of early CC and gastric cancer (Liu et al., 2020; Liu H. et al., 2019; Barbagallo et al., 2018; Dai et al., 2017; Zhao et al., 2015). After comparing AUC values, we believe that FEZF1-AS1 has a high diagnostic value for CC. According to the study report, in patients with gastric cancer, the sensitivity and specificity of FEZF1-AS1 are found to be higher than those of conventional tumor markers (such as CEA) when analyzing AUC, and the detection of serum FEZF1-AS1 and CEA can improve the diagnostic sensitivity of gastric cancer (Liu et al., 2020). The survival analysis of Bian et al. (2018) showed that high expression of FEZF1-AS1 significantly leads to poor prognosis in CC patients. To delve deeper into the prognostic impact of FEZF1-AS1, both univariate and multivariate studies are conducted, revealing FEZF1-AS1 as an independent prognostic factor for CC. However, our COX regression and survival study reveal no link between FEZF1-AS1 and the survival rates of CC patients. This result may be due to the fact that the number of CC patients is still not large enough. Therefore, we need to expand the sample size to further analyze the relationship between FEZF1-AS1 and the survival of CC patients.

Bian et al. (2018) have confirmed that FEZF1-AS1 can enhance cell growth, migration, and invasion in CC and inhibit apoptosis of tumor cells. It is consistent with our findings. It shows that the activation of autophagy can inhibit the apoptosis of CC cells and promote their proliferation (Liu J. et al., 2019). In our study, according to the TCGA database and RNA-seq, FEZF1-AS1 is functionally enriched into autophagy and the PI3K/AKT signaling pathway. We conclude that FEZF1-AS1 promotes CC progression through autophagy. In recent years, some studies have reported that abnormal lncRNAs participate in the chemoresistance of some tumors, including CC, by regulating mRNA transcription, translation, and protein stability (Holohan et al., 2013; Yuan et al., 2016; Zhang C-L. et al., 2017). Moreover, an enhanced autophagy level was detected in patients with chemoresistance with poor prognosis, suggesting that the presence of autophagy may promote the development of chemoresistance (Shuhua et al., 2015). It is reported that FEZF1-AS1 promotes the development of chemoresistance in gastric cancer cells by upregulating ATG5 (Gui et al., 2021). At present, no studies have reported whether FEZF1-AS1 produces chemoresistance on CC cells by mediating autophagy. In our study, we initially explored the effect of FEZF1-AS1 on Oxa sensitivity of CC cells. The result shows that FEZF1-AS1 decreased the sensitivity of CC cells to Oxa by mediating autophagy. However, for chemoresistance, it is still necessary to culture CC-resistant strains for detection.

Numerous studies have shown that autophagy involves the PI3K/AKT/mTOR signaling pathway (Janku et al., 2011; Heras-Sandoval et al., 2014). Su et al. (2015) believed that the PI3K/AKT/mTOR pathway plays a crucial role in inhibiting autophagy to control tumor growth. Zheng et al. (2019) confirmed that the PI3K/AKT/mTOR signaling pathway encompasses signal transmission, the movement of autophagosomes, and the merging of vesicles during autophagy. Inhibition of AKT to induce autophagy and activation of AKT to inhibit autophagy have also been observed in some studies (Sun et al., 2013; Roy et al., 2014). Therefore, regulating the PI3K/AKT/mTOR signaling pathway is crucial for preserving the balance in autophagy. A study has determined that GOLPH3 promotes Oxa resistance in CC cells through the PI3K/AKT/mTOR signaling pathway (Yu et al., 2020). El-Daly et al. (2023) demonstrated that inhibiting the activity of the PI3K/AKT/mTOR signaling pathway increases the chemotherapy sensitivity of CC cells to 5-fluorouracil. These results all suggest that the PI3K/AKT/mTOR signaling pathway is involved in the chemotherapy resistance process of CC. In our study, WB and IF are used to detect the PI3K/AKT/mTOR signaling pathway and key autophagy proteins (LC3 and P62), and autophagy inhibitors and mTOR inhibitors are used for rescue experiments. We find that FEZF1-AS1 activates autophagy by inhibiting the PI3K/AKT/mTOR signaling pathway, thereby promoting the development of CC. In general, there is no alteration in the expression levels of the total PI3K, AKT, and mTOR proteins. When the pathway is activated, the protein is phosphorylated and activated to transmit signals downstream. Our research indicates alterations in the overall levels of PI3K, AKT, and mTOR proteins within the PI3K/AKT/mTOR signaling pathway, suggesting that FEZF1-AS1 may directly interact with PI3K, AKT, and mTOR to change their protein expression, and then activate autophagy.

FEZF1-AS1 has demonstrated a significant value in oncology, precision medicine, and disease mechanism research in recent years, especially having potential translational significance in tumor diagnosis, treatment, and prognosis evaluation. In our study, FEZF1-AS1, as a novel tumor biomarker, has a high value in aspects such as the early diagnosis of tumors and the potential for noninvasive detection. Meanwhile, FEZF1-AS1, as a potential therapeutic target, is expected to guide individualized treatment.

The innovation of this study is to find FEZF1-AS1 as the target molecule through bioinformatics. First, in CC, FEZF1-AS1 promotes the development of CC and reduces the sensitivity of CC cells to Oxa by activating autophagy. Second, FEZF1-AS1 activates autophagy through the PI3K/AKT/mTOR signaling pathway. Next, we will expand the sample size of CC and analyze the influence of FEZF1-AS1 on the prognosis of CC patients. Based on the results of RNA-seq and mass spectrometry, the upstream mechanisms related to FEZF1-AS1, such as transcription factors and regulatory elements, will be explored. The direct molecular effects will be verified through RNA pull down and RNA immunoprecipitation techniques. We will culture CC chemoresistance cell lines and further study the relationship between FEZF1-AS1 and CC chemoresistance.

## Conclusion

FEZF1-AS1 drives autophagy-mediated development of CC and reduces chemosensitivity through inhabiting the PI3K/AKT/mTOR signaling pathway. In conclusion, FEZF1-AS1 is expected to be a biomarker for the diagnosis, progression, and efficacy evaluation of CC.

## Data Availability

The raw data supporting the conclusions of this article will be made available by the authors, without undue reservation.

## Glossary

CC: colon cancer
lncRNA: long non-coding RNA
FEZF1-AS1: FEZ family zinc finger 1-antisense RNA 1
mRNA: messenger RNA
ceRNA: competitive endogenous RNA
miRNA: microRNA
ATG: autophagy-related gene
LC3: microtubule-associated protein 1 light chain 3
TCGA: The Cancer Genome Atlas
RNA-seq: RNA sequencing
logFC: log2 fold change
P. Val: corrected P-value
GO: Gene Ontology
KEGG: Kyoto Encyclopedia of Genes and Genomes
DEmRNA: differentially expressed mRNA
ROC: receiver operating characteristic
AUC: area under curve
qRT-PCR: real-time quantitative PCR
CCK8: Cell Counting Kit-8
OD: absorbance
WB: Western blotting
IF: immunofluorescence
IHC: immunohistochemistry
CQ: chloroquine
RA: rapamycin
Oxa: oxaliplatin
DElncRNA: differentially expressed lncRNA
ABC: ATP-binding cassette
ROS: reactive oxygen species
OS: overall survival
DFS: disease-free survival
DEmiRNA: differentially expressed miRNA.
