# Correction: FEZF1-AS1 drives autophagy-mediated progression of colon cancer and reduces chemosensitivity through inhabiting the PI3K/AKT/mTOR signaling pathway

**DOI:** 10.3389/fgene.2025.1684479

**Published:** 2025-10-07

**Authors:** Xiaoping Yang, Zuohui Yuan, Lingzhu Gou, Long Cheng, Zirui Wang, Pingfan Wu, Xiaochun Wang, Xueni Ma, Tiantian Ma, Yi Yu, Zhiping Wu, Dekui Zhang

**Affiliations:** ^1^ Key Laboratory of Digestive Diseases of Gansu Province, The Second Hospital and Clinical Medical School, Lanzhou University, Lanzhou, China; ^2^ Department of Gastroenterology, Gansu Provincial Hospital, Lanzhou, China; ^3^ Department of Urology, The Second People’s Hospital of Lanzhou, Lanzhou, China; ^4^ Department of Pathology, The Sixth Affiliated Hospital, School of Medicine, South China University of Technology, Foshan, China; ^5^ Department of Pneumology, Xi An Chest Hospital, Xian, China; ^6^ Department of Gastroenterology, The Second Hospital and Clinical Medical School, Lanzhou University, Lanzhou, China

**Keywords:** FEZF1-AS1, colon cancer, autophagy, PI3K/AKT/mTOR, chemosensitivity

Affiliations 1 and 2 were numbered in the wrong order.

The original version of this article has been updated.

