# Supplementary material for: FEZF1-AS1 drives autophagy-mediated progression of colon cancer and reduces chemosensitivity through inhabiting the PI3K/AKT/mTOR signaling pathway
Source: Front Genet. 2025 Jul 24;16:1514205. doi: 10.3389/fgene.2025.1514205 (PMC12328158; doi:10.3389/fgene.2025.1514205)
Supplement: Supplementary file 1 [file DataSheet1.pdf]

Table S1. Primer sequences of qRT-PCR

Gene name	Primer sequences
FEZF1-AS1-F	TATGACTCAGGGTTGGACTTTATGG
FEZF1-AS1-R	TTGTTGCTTGACGCTGTTGCT
β-actin-F	TGGCACCCAGCACAATGAA
β-actin-R	CTAAGTCATAGTCCGCCTAGAAGCA

Table S2. Interference targets and the synthesis of primers of target fragments

Gene name	sequences
NC siRNA	TTCTCCGAACGTGTCACGTAA
FEZF1-AS1 siRNA	GCTTCCGAGTTTCCATTGA
LV-h-FEZF1-AS1-E/B-F	CTACTAGAGGATCTATTTCCGGTGAATTCGA AAACTTTGGGCTTGGCAT
LV-h-FEZF1-AS1-E/B-R	GATCCTTACTAGTATCGATGGATCCAATTTC TATTATCGACTTTTATTT

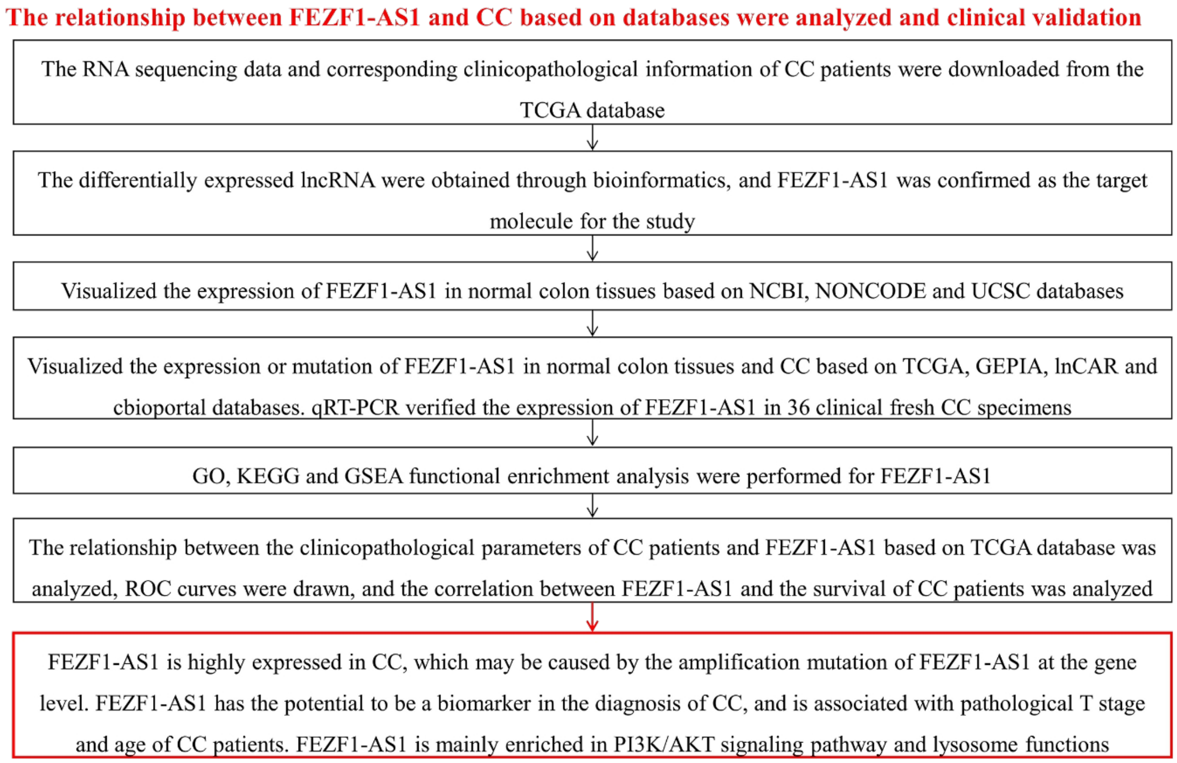
The relationship between FEZF1-AS1 and CC based on databases were analyzed and clinical validation
The RNA sequencing data and corresponding clinicopathological information of CC patients were downloaded from the TCGA database
The differentially expressed lncRNA were obtained through bioinformatics, and FEZF1-AS1 was confirmed as the target molecule for the study
Visualized the expression of FEZF1-AS1 in normal colon tissues based on NCBI, NONCODE and UCSC databases
Visualized the expression or mutation of FEZF1-AS1 in normal colon tissues and CC based on TCGA, GEPIA, lnCAR and cbioportal databases. qRT-PCR verified the expression of FEZF1-AS1 in 36 clinical fresh CC specimens
GO, KEGG and GSEA functional enrichment analysis were performed for FEZF1-AS1
The relationship between the clinicopathological parameters of CC patients and FEZF1-AS1 based on TCGA database was analyzed, ROC curves were drawn, and the correlation between FEZF1-AS1 and the survival of CC patients was analyzed
FEZF1-AS1 is highly expressed in CC, which may be caused by the amplification mutation of FEZF1-AS1 at the gene level. FEZF1-AS1 has the potential to be a biomarker in the diagnosis of CC, and is associated with pathological T stage and age of CC patients. FEZF1-AS1 is mainly enriched in PI3K/AKT signaling pathway and lysosome functions

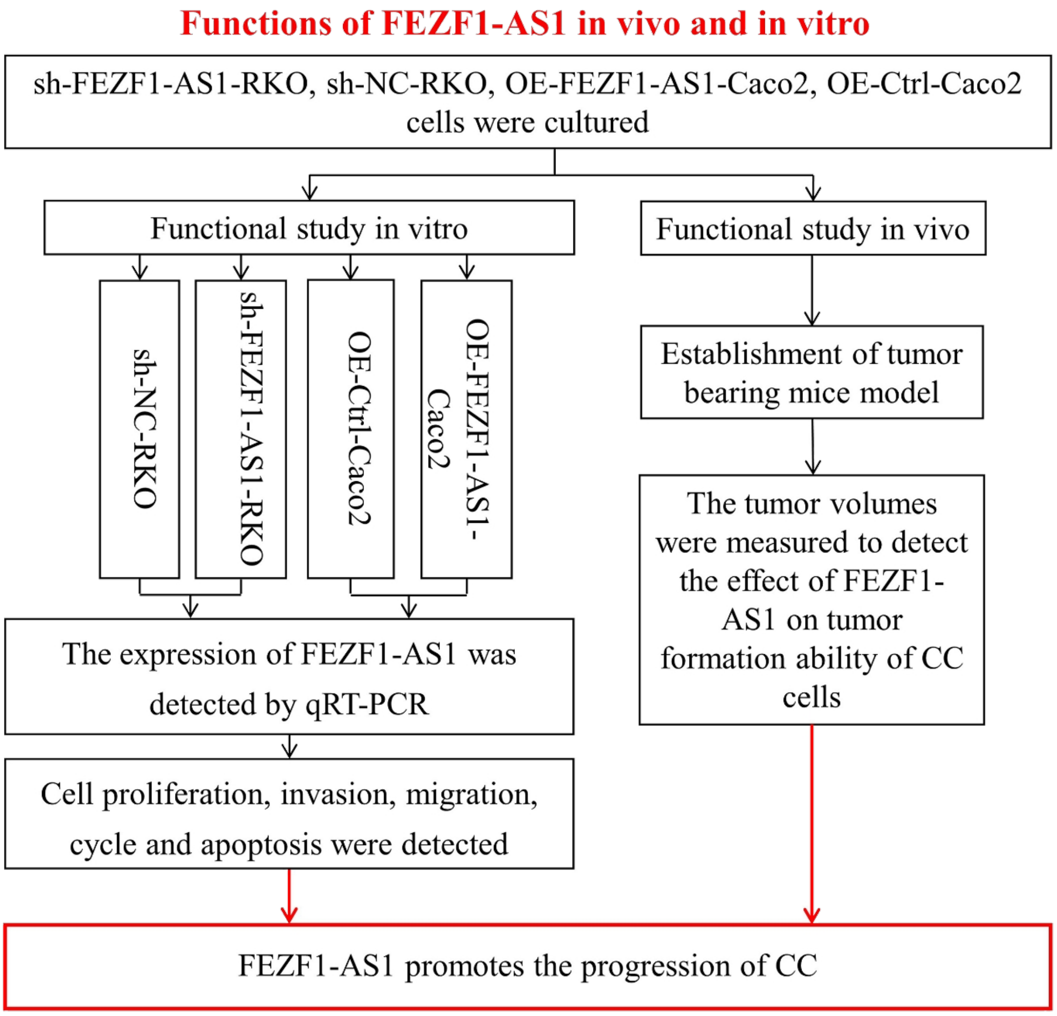
Functions of FEZF1-AS1 in vivo and in vitro
sh-FEZF1-AS1-RKO, sh-NC-RKO, OE-FEZF1-AS1-Caco2, OE-Ctrl-Caco2 cells were cultured
Functional study in vitro
Functional study in vivo
sh-NC-RKO
sh-FEZF1-AS1-RKO
OE-Ctrl-Caco2
OE-FEZF1-AS1-Caco2
Establishment of tumor bearing mice model
The tumor volumes were measured to detect the effect of FEZF1-AS1 on tumor formation ability of CC cells
The expression of FEZF1-AS1 was detected by qRT-PCR
Cell proliferation, invasion, migration, cycle and apoptosis were detected
FEZF1-AS1 promotes the progression of CC

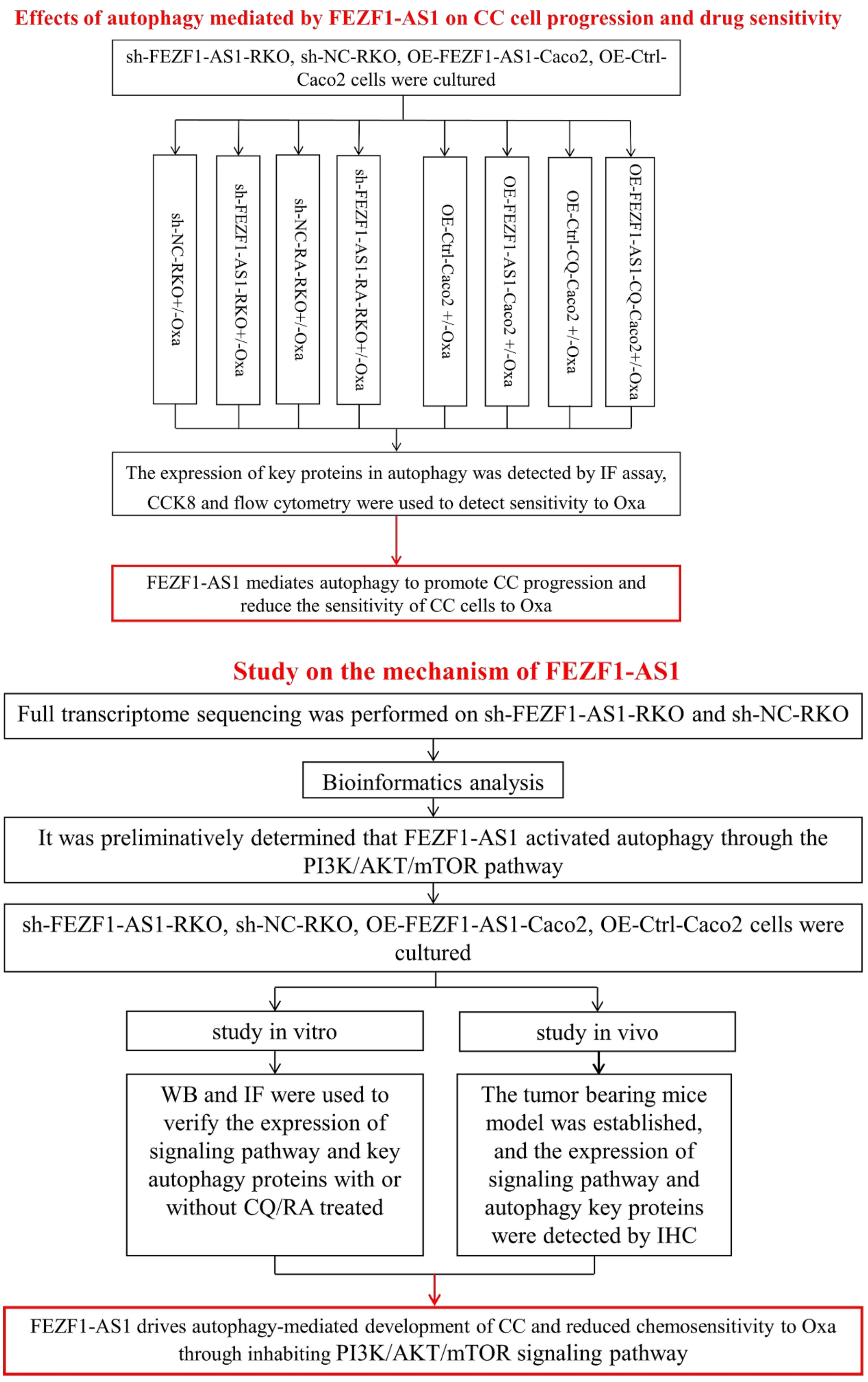

Fig.S1 The technology roadmaps of manuscript.

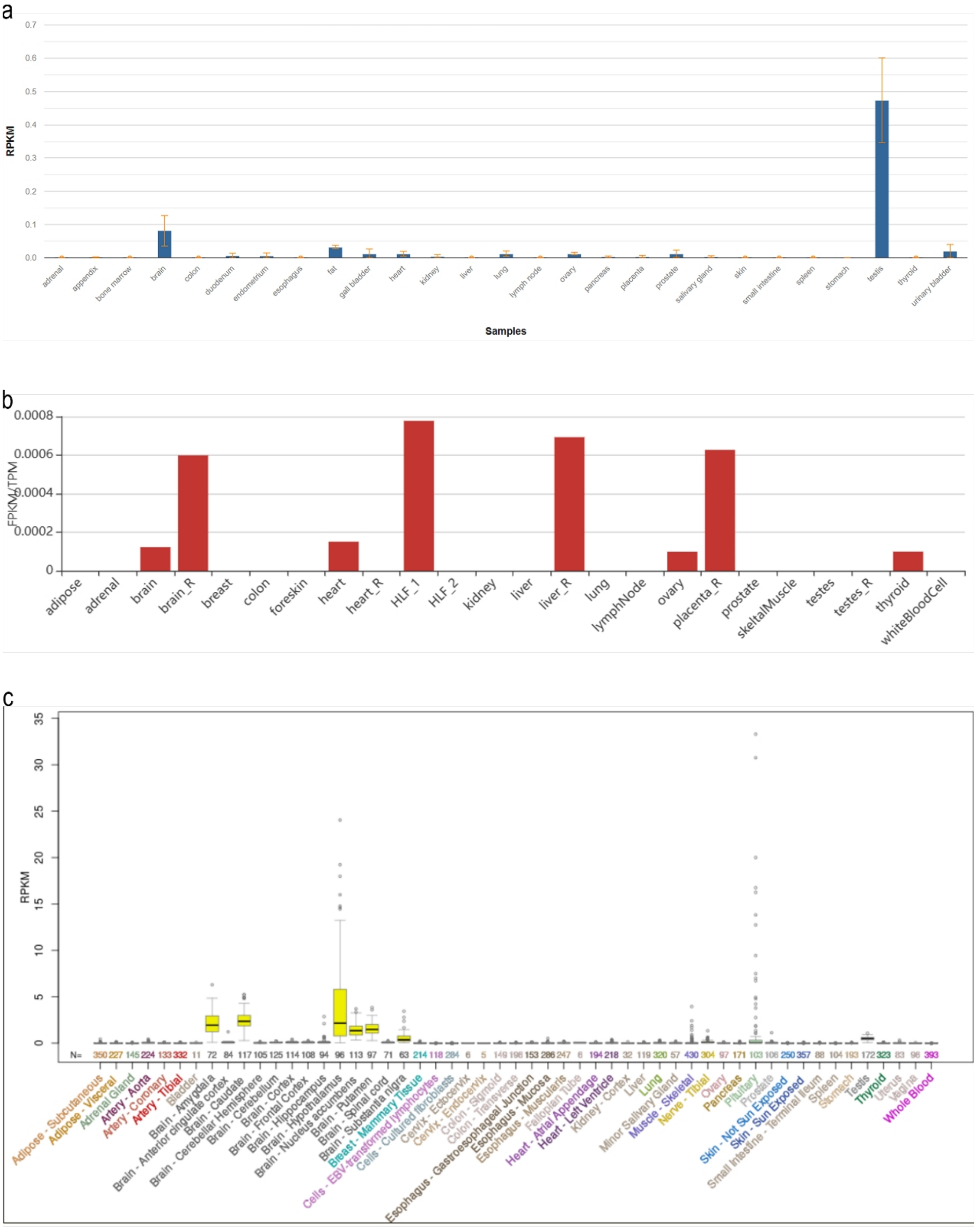

Fig.S2 FEZF1-AS1 expression was upregulated in CC tissues. In (a) NCBI, (b) NONCODE and (c) UCSC databases, FEZF1-AS1 was in a low expression pattern in normal colon tissues.

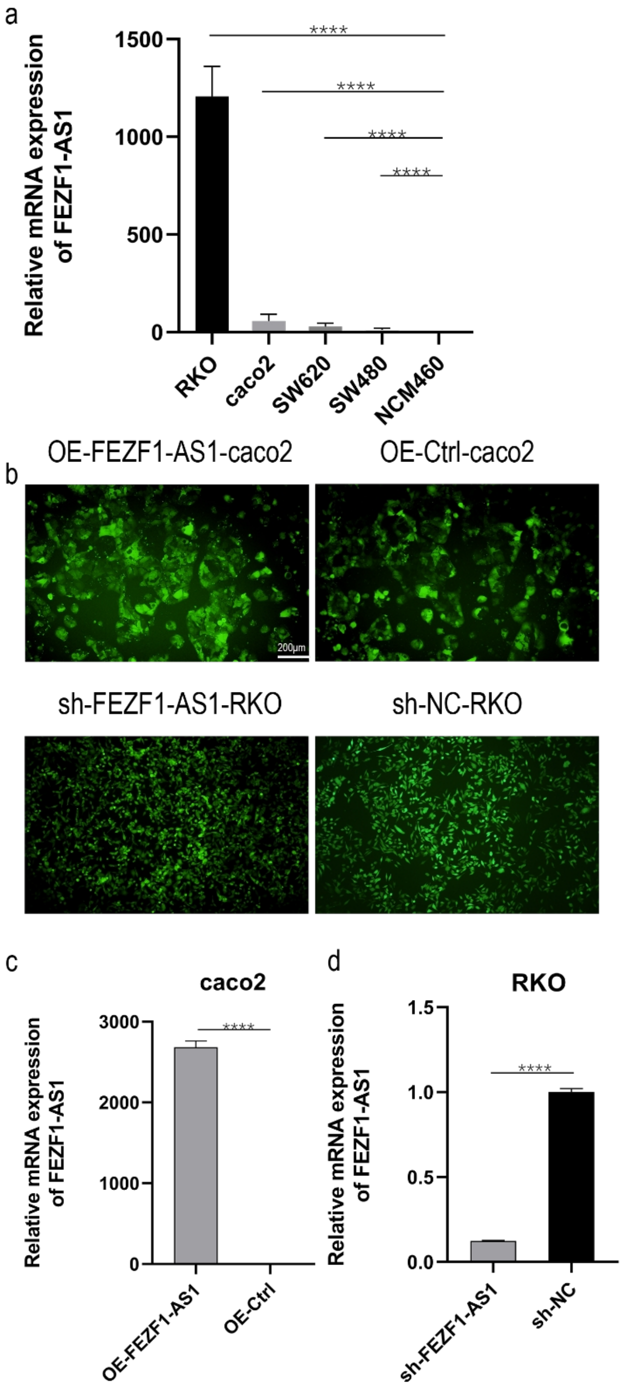

Fig.S3 Construction of lentivirus cell lines with FEZF1-AS1 overexpression and knockdown. (a) qRT-PCR was used to identify the expression of FEZF1-AS1. RKO, caco2, SW620, SW480 were CC cells, and NCM460 was normal epithelial cell of colon. (b) Lentiviral fluorescence transfection efficiency of cells. (c) Efficiency of FEZF1-AS1 overexpression in cell. (d) Efficiency of FEZF1-AS1 knockdown in cell. ****, $P < 0.0001$.

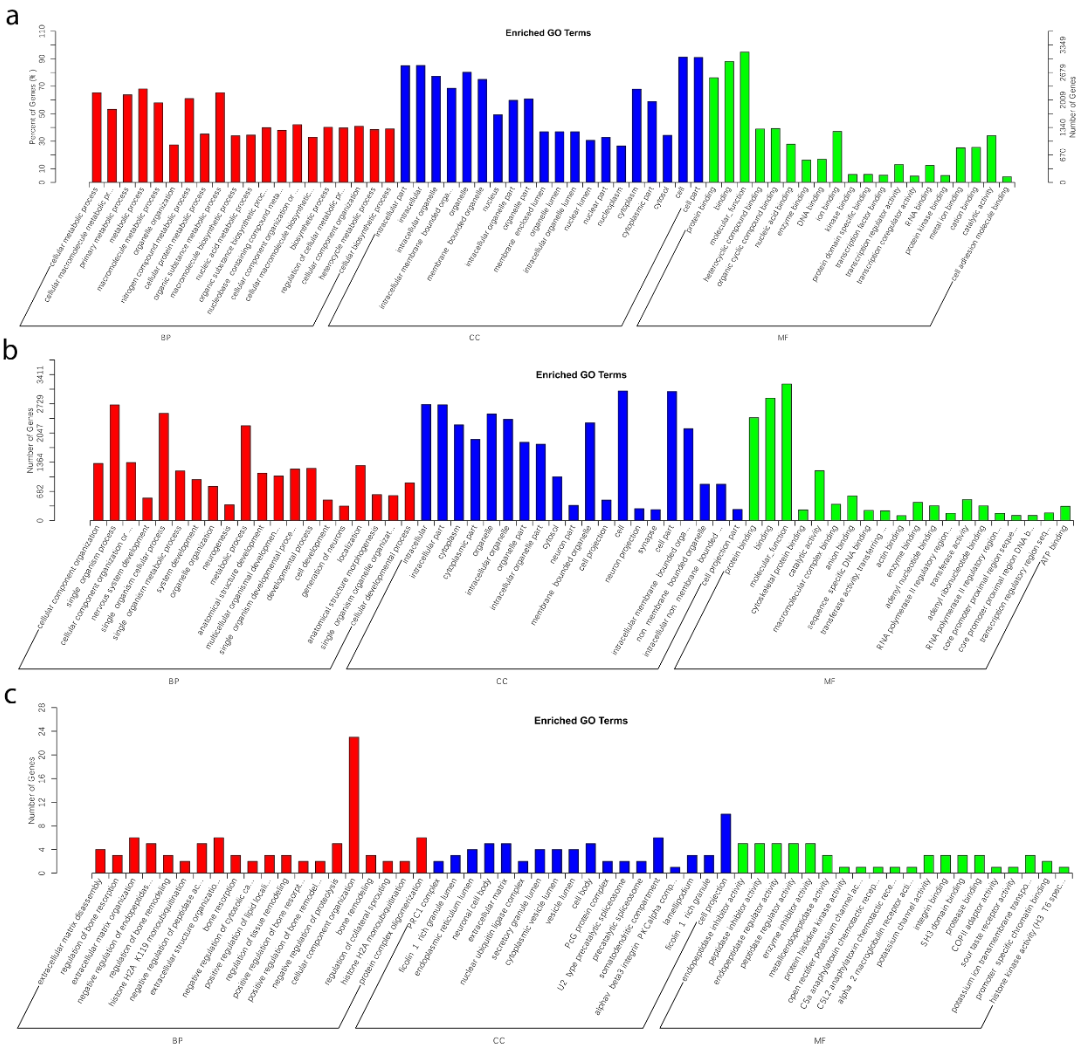

Fig.S4 GO enrichment analysis. (a) GO functional enrichment analysis of FEZF1-AS1 and its co-expressed gene set in sh-FEZF1-AS1-RKO cell and sh-NC-RKO cell. (b) GO functional enrichment analysis of DEmiRNA and its co-expressed gene set in sh-FEZF1-AS1-RKO cell and sh-NC-RKO cell. (c) GO functional enrichment analysis of DEmRNA and its co-expressed gene set in sh-FEZF1-AS1-RKO cell and sh-NC-RKO cell. BP, Biological Process, CC, Cellular Component, and MF, Molecular Function.
